# The *Medicago truncatula* Vacuolar iron Transporter‐Like proteins VTL4 and VTL8 deliver iron to symbiotic bacteria at different stages of the infection process

**DOI:** 10.1111/nph.16735

**Published:** 2020-07-16

**Authors:** Jennifer H. Walton, Gyöngyi Kontra‐Kováts, Robert T. Green, Ágota Domonkos, Beatrix Horváth, Ella M. Brear, Marina Franceschetti, Péter Kaló, Janneke Balk

**Affiliations:** ^1^ Department of Biological Chemistry John Innes Centre Norwich NR4 7UH UK; ^2^ School of Biological Sciences University of East Anglia Norwich NR4 7TJ UK; ^3^ Agricultural Biotechnology Institute NARIC Gödöllő 2100 Hungary; ^4^ School of Life and Environmental Sciences The University of Sydney Sydney NSW 2006 Australia; ^5^ Institute of Plant Biology Biological Research Centre Szeged 6726 Hungary

**Keywords:** iron, *Medicago*, micronutrient, nitrogen fixation, nodulin‐21, symbiosis, vacuole

## Abstract

- The symbiotic relationship between legumes and rhizobium bacteria in root nodules has a high demand for iron, and questions remain regarding which transporters are involved. Here, we characterize two nodule‐specific Vacuolar iron Transporter‐Like (VTL) proteins in *Medicago truncatula*.
- Localization of fluorescent fusion proteins and mutant studies were carried out to correlate with existing RNA‐seq data showing differential expression of *VTL4* and *VTL8* during early and late infection, respectively.
- The *vtl4* insertion lines showed decreased nitrogen fixation capacity associated with more immature nodules and less elongated bacteroids. A mutant line lacking the tandemly‐arranged *VTL4*–*VTL8* genes, named 13U, was unable to develop functional nodules and failed to fix nitrogen, which was almost fully restored by expression of *VTL8* alone. Using a newly developed *lux* reporter to monitor iron status of the bacteroids, a moderate decrease in luminescence signal was observed in *vtl4* mutant nodules and a strong decrease in 13U nodules. Iron transport capability of VTL4 and VTL8 was shown by yeast complementation.
- These data indicate that VTL8, the closest homologue of SEN1 in *Lotus japonicus*, is the main route for delivering iron to symbiotic rhizobia. We propose that a failure in iron protein maturation leads to early senescence of the bacteroids.

## Introduction

Legumes and a small number of other plant species (*Parasponia* sp.) are able to form a symbiosis with rhizobium bacteria which enables the host plant to access N_2_ as a source of nitrogen. The host plant provides carbohydrates derived from photosynthesis for the energy‐demanding reduction of N_2_ to ammonium, carried out by the bacterial nitrogenase enzyme. Successful establishment of the symbiosis in specialized structures called root nodules depends on signalling and recognition between the rhizobia and the host plant, as well as later checkpoints during nodule development (reviewed in Oldroyd *et al*., 2011; Suzaki *et al*., 2015).

Root nodules have a high requirement for iron owing to abundant proteins that use iron as a cofactor. Infected plant cells produce large amounts of haem for leghaemoglobins, which maintain a microaerobic environment for the oxygen‐sensitive nitrogenase enzyme (Downie, 2005; Ott *et al*., 2005; Garrocho‐Villegas *et al*., 2007). The bacterial nitrogenase enzyme (NifH + NifDK) binds 12 iron in the form of iron–sulphur clusters and another seven in the iron–molybdenum cofactor (Dean *et al*., 1993; Howard & Rees, 2006). The nitrogen‐fixing bacteria also contain numerous cytochromes and other iron proteins. When the plant is starved of iron, nodule initiation and further development is strongly impaired (O’Hara *et al*., 1988; Tang *et al*., 1990; Brear *et al*., 2013).

Nodule development is initiated by a signalling cascade between plant roots and the bacteria, entrapment of the bacteria in curled root hairs and formation of infection threads along which the bacteria travel into the root cortex. Division of root cortical cells leads to formation of the nodule, considered a specialized root outgrowth. The nodules formed by *Medicago truncatula* in symbiosis with *Sinorhizobium meliloti* or *S. medicae* are of the indeterminate type. The meristem persists over time and generates a gradient of cells at progressing developmental stages (Vasse *et al*., 1990; Roux *et al*., 2014), commonly divided into four histological zones, with the meristem as Zone I. Zone II corresponds to the infection zone where bacteria are released from the infection threads, divide and form symbiosomes – intracellular bacteria surrounded by a plant‐derived membrane. In older cells of Zone II, bacterial cell division stops while genome replication continues, resulting in elongated polyploid bacteroids (Mergaert *et al*., 2006). Synchrotron X‐ray fluorescence (XRF) studies on *M. truncatula* indicated that iron is delivered to Zone II by the vascular bundles in the nodule (Rodríguez‐Haas *et al*., 2013). In the latter study ‘iron‐rich bodies’ were observed in and near the vascular bundles which may correspond to the iron storage protein ferritin, the expression of which is induced in Zone II (Strozycki *et al*., 2007; Roux *et al*., 2014). In the Interzone (transition of Zone II to III), infected plant cells and the bacteroids complete their differentiation with the last rounds of endoreduplication and enlargement, resulting in a striking cell morphology of tightly packed elongated rhizobia surrounding a central vacuole. Zone III comprises the major part of a functional nodule and this is where nitrogen‐fixation takes place. In older nodules, a zone of senescent cells, Zone IV, is formed from which nutrients such as iron are recycled from degraded plant cells and symbionts (Van de Velde *et al*., 2006; Rodríguez‐Haas *et al*., 2013).

Forward and reverse genetic studies have identified several transporters involved in iron delivery to the nodules and symbiosomes. The closely related genes *Lotus japonicus MATE1* and *M. truncatula MATE67* are highly induced during nodule development, specifically in the infection zone (Takanashi *et al*., 2013; Wang *et al*., 2017; Kryvoruchko *et al*., 2018). Studies in *Xenopus* oocytes showed that the LjMATE1 and MtMATE67 proteins can transport citrate, an organic chelator of Fe^3+^ in the xylem (Takanashi *et al*., 2013; Kryvoruchko *et al*., 2018). The MtMATE67 protein is localized to vascular bundles but also to the symbiosome membrane (Kryvoruchko *et al*., 2018). An RNAi mutant of *LjMATE1* accumulated high levels of iron in the vascular bundle of roots and nodules, whereas iron staining was fainter in the infection zone of mutant nodules, particularly in the central region, compared to wild‐type (Takanashi *et al*., 2013). These two studies indicate that the host plant delivers iron to the nodules via the xylem.

Uptake of iron into the infected cells may involve *MtNRAMP1*, one of seven members of this gene family in *M. truncatula* with the highest relative expression in roots and nodules (Tejada‐Jiménez *et al*., 2015). Yeast complementation studies showed that MtNRAMP1 can transport iron and manganese, similar to its closest Arabidopsis homologue which is the primary route for manganese into the cell (Cailliatte *et al*., 2010). MtNRAMP1 is localized to plasma membranes, including the plasma membrane of infected cells. A transposon insertion mutant of *MtNRAMP1* has impaired nodule development, but still exhibited 60% nitrogenase activity compared to wild‐type (Tejada‐Jiménez *et al*., 2015).

**Fig. 1:**
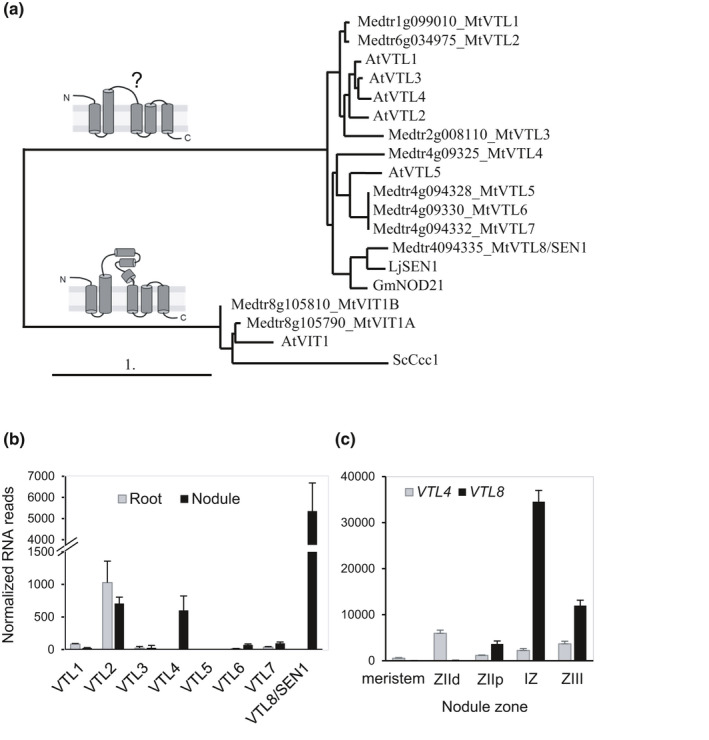
Vacuolar iron Transporter‐Like (VTL) proteins in *Medicago truncatula*. (a) Phylogenetic relationship and predicted domain structure of VTL protein sequences identified in the *M. truncatula* genome and those previously reported in *Arabidopsis thaliana* (*AtVTL1*,* AT1G21140*; *AtVTL2*, *AT1G76800*; *AtVTL3*,* AT3G43630*; *AtVTL4*,* At3G43660*; *AtVTL5*, *AT3G25190*). The ‘true’ Vacuolar Iron Transporter (VIT) sequences including Ccc1 from *Saccharomyces cerevisiae* served as an outgroup. The domain structure of VIT proteins is adapted from Kato *et al*. (2019). (b, c) Expression of *M. truncatula VTL* genes in roots and nodules (b) and in different nodule zones (c). Data were obtained from the Symbimics website (https://iant.toulouse.inra.fr/symbimics), and are Ribominus‐like values for (b) and DESeq‐normalized reads from laser‐capture microdissection fractions for (c). ZIId and ZIIp, distal and proximal fractions of Zone II, respectively. IZ, Interzone. ZIII, nitrogen‐fixing Zone III. Error bars represent SE.

For iron to reach the bacteroid, it needs to be exported by the infected plant cell and imported across the bacteroid membrane. Five different allelic mutants in the *LjSEN1* gene, encoding a Vacuolar iron Transporter‐Like (VTL) protein, were identified in a screen for ineffective symbiotic (Fix‐) mutants in *L. japonicus* (Hakoyama *et al*., 2011). Promoter‐GUS studies showed that *LjSEN1* is expressed exclusively in rhizobia‐infected cells, but its subcellular localization and function in iron transport has not been documented to date. The VTL proteins differ from the Vacuolar Iron Transporter (VIT) proteins in that the former lack a cytosolic loop thought to mediate Fe^2+^/H^+^ antiport in VIT, see diagrams in Fig. 1a (Labarbuta *et al*., 2017; Kato *et al*., 2019). VIT proteins are well characterized in yeast, plants and *Plasmodium*, mediating iron transport into the vacuole for detoxifying excess iron, or for building up iron stores in seeds (Kim *et al*., 2006; Kato *et al*., 2019). In contrast, the physiological function of VTLs is less clear, although expression patterns and functional studies in Arabidopsis suggest that VTLs also play a role in iron homeostasis (Gollhofer *et al*., 2011).

To better understand the role of VTLs in nodule development and biological nitrogen fixation, we characterized two *VTL* genes in *M. truncatula*, *VTL4* and *VTL8*, which are specifically expressed in nodules. Mutant studies showed they play a role at early and late stages, respectively, of nodule development. In particular VTL8 is critical for delivering iron to the bacteroids and for establishment of the nitrogen‐fixing bacteria. While this article was undergoing review, two studies describing a specific VTL in soybean nodule development were also accepted for publication (Brear *et al*., 2020; Liu *et al*., 2020).

## Materials and Methods

### Plant lines and growth

*Medicago truncatula* Jemalong and *M. truncatula* subsp. *tricycla* R108 genotypes were used as wild‐type controls for the 13U and *vtl4* mutant lines, respectively. The *Tnt1*‐insertion mutants *vtl4‐1* (NF17463) and *vtl4‐2* (NF21016) were identified by reverse screening using PCR and purchased from the Samuel Roberts Noble Foundation (Ardmore, OK, USA). These lines have approximately 25 insertions each, and a list of the positions of all known insertions is given in Supporting Information Table S1. While most insertions are outside of annotated genes, the 3–5 insertions that could potentially affect other genes (if homozygous) are not specifically expressed in nodules, except for *VTL4* (Limpens *et al*., 2013; Roux *et al*., 2014). Primer sequences for genotyping *vtl4* mutants are listed in Table S2.

The 13U mutant line was isolated from a collection of fast neutron radiation mutants (Domonkos *et al*., 2013) and the deletion was identified using a combined approach of genetic mapping and microarray‐based Affymetrix GeneChip hybridization (Murray *et al*., 2011). The boundaries of the deletion were determined by PCR, see Table S2 for primer sequences.

Seeds were scarified, surface sterilized with 10% (*w/v*) sodium hypochlorite, stratified at 4°C for 3 to 7 d and then germinated at 20°C. The next day, seedlings were plated out on modified Fahraeus medium (0.7 mM KH_2_PO_4_, 0.8 mM Na_2_HPO_4_, 0.50 mM MgSO_4_, 0.7 mM CaCl_2_, 20 µM Fe‐citrate, pH 6.0), and micronutrients (5 µM H_3_BO_3_, 9 µM MnSO_4_, 0.8 µM ZnSO_4_, 0.3 µM CuSO_4_ and 0.5 µM H_2_MoO_4_), or planted out on a 1 : 1 mixture of Terragreen–sand, or on zeolite (Geoproduct Kft., Mád, Hungary), as indicated in figure legends. Plants were grown in long‐day conditions (16 h : 8 h, light : dark) at 22°C, light intensity 180–200 μmol photons m^−2^ s^−1^.

### Bacterial strains and growth

Two different bacterial strains were used for nodulating *M. truncatula*, as indicated in the figure legends. *Sinorhizobium* (*Ensifer*) *medicae* WSM419 (pXLGD4 P*hemA*:*lacZ*,* tetR*) was generally used for nodulating *M. truncatula* Jemalong and the 13U mutant; *S. meliloti* 1021 (P*nifA:lacZ*, *tetR*) was used for *M. truncatula* subsp. *tricycla* R108 and the *vtl4* mutants. Bacteria were grown in TY medium (1% (w/v) tryptone, 0.3% (w/v) yeast extract, 6 mM CaCl_2_), either in liquid medium for inoculation or on solid medium with 1% (w/v) agar for maintaining the strains. For testing iron‐dependent regulation of P*mbfA:lux*, *Rhizobium leguminosarum* bv. *viciae* strain J251 was used as wild‐type and its derivative J386 carrying a Tn5::*lacZ* insertion in *irr* (Wexler *et al*., 2003).

### *Agrobacterium rhizogenes*‐mediated complementation of *M. truncatula* mutants

DNA fragments containing the *VTL4* and *VTL8* genes including the 1.5 and 1.6 kb promoter sequences and 0.9 and 1.2 kb 3′‐untranslated regions, respectively, were amplified with oligonucleotides (see Table S2) from BAC (bacterial artificial chromosome) clone mth2‐28d20. The destination vector pKGW‐R (www.gateway.psb.ugent.be/vector/show/pKGW_RedRoot/) was linearized with restriction enzymes *Aat*II and *Spe*I. The gene fragments and the linearized vector were fused using the In‐Fusion HD Cloning Kit (Clontech Laboratories/Takara Bio, Mountain View, CA, USA) according to the manufacturer's protocol. Constructs were introduced into the ARqua1 strain of *Agrobacterium rhizogenes* and used for hairy root transformation as described in The *Medicago truncatula* Handbook (Chabaud *et al*., 2006). Transformed roots were identified by fluorescence of the DsRed marker protein.

### Protein localization and fluorescence microscopy

The *VTL4* promoter (2.5 kb), *VTL8* promoter (2.1 kb) and coding sequences were domesticated for Golden Gate assembly. A glycine‐rich linker sequence (Table S2) was added to the C‐terminus followed by the coding sequence of mCherry. As a marker for membranes, the *Arabidopsis thaliana PIP2A* (*AT3G53420*) sequence was fused to eGFP and placed behind the promoter of *L. japonicus UBIQUITIN* (GenBank DQ249171.1) The two sequences were assembled together into vector pL1‐R1 and transformed into *Agrobacterium rhizogenes* ARqua1 for hairy root transformation of *M. truncatula* R108. Nodules were harvested after 21 d and imaged using a Leica TCS SP5 confocal microscope with standard settings for eGFP, mCherry and DAPI (4′,6‐diamidino‐2‐phenylindole). To analyse nodule occupancy by rhizobia, nodule sections were stained with the DNA‐binding fluorescent dye SYTO13 (Invitrogen, Eugene, OR, USA) as previously described (Domonkos *et al*., 2013).

### P*mbfA:lux* reporter and activity assays

Nucleotides −487 to +104, containing the promoter sequence and part of the coding sequence of the *mbfA* gene (SMc00359) were PCR‐amplified from *S. meliloti* 1021 (see Table S2 for primer sequences) and cloned using the *Bam*HI and *Kpn*I restriction sites into pIJ11268 upstream of the *lux* operon (Frederix *et al*., 2014). A de‐repressed version of the reporter was made by mutating the Iron Control Element (ICE) from TTCTAA to AGCTTC (−19 to −14) by site‐directed mutagenesis. The pIJ11282 plasmid expressing *lux* constitutively has been described previously (Frederix *et al*., 2014). The plasmids were transferred from *Escherichia coli* to *S. meliloti* or *R. leguminosarum* by conjugation using an *E. coli* helper strain carrying plasmid pRK2013. For luminescence assays, overnight bacterial cultures were diluted in UMS medium (Wheatley *et al*., 2017) without or with 40 µM iron sulphate (FeSO_4_) as indicated. Luminescence and OD_600_ of triplicate cultures were measured in a ClarioStar microplate reader (BMG Labtech, Ortenberg, Germany).

### Luminescence imaging and quantification

Plants growing on Terragreen–sand mixture were inoculated with *S. meliloti* 1021 carrying the P*mbfA:lux* reporter plasmid or the mutated P*mbfA^ICE^:lux* reporter at 7 d post‐germination, and grown for a further 21 d. Plants were dug up, roots rinsed with distilled water, blotted dry and imaged using the NightOWL II LB 983 *in vivo* imaging system with IndiGO software (Berthold Technologies, Bad Wildbad, Germany). Luminescent nodules were identified using the automated peak picking tool. Luminescence values in photons per square millimetre were calculated from the output. At least five plants per line were analysed with each inoculum.

### Miscellaneous methods

For details on gene expression analysis and yeast complementation assays, see Methods S1. Acetylene reduction assays were performed as previously described (Domonkos *et al*., 2013). Purified bacteroids were stained with propidium iodide to determine their length using confocal microscopy and imagej software. Protein blot analysis with antibodies against *Pisum sativum* ferritin and Arabidopsis hemoglobin 2 (Agrisera, Vännas, Sweden) was carried out following supplier's protocols. The iron concentration of nodules was determined photospectrometrically using the colorimetric chelator ferene (3‐(2‐pyridyl)‐5,6(5‐sulfo‐2‐furyl)‐1,2,4‐triazine) on mineralized tissue samples. Iron staining of nodules was performed using the Perls’ method (Meguro *et al*., 2007).

## Results

### *Medicago truncatula* has eight *VTL* genes of which two are specifically expressed in nodules

The *M. truncatula* genome (Mt4.0v1) was searched for homologues of *Arabidopsis thaliana VIT* and *VTL* genes. We identified two homologues of *VIT* and eight homologues of *VTL* as compared to one and five genes, respectively, in *Arabidopsis thaliana*. Amino acid alignment of the eight MtVTLs with the *Arabidopsis thaliana* homologues showed only a limited phylogenetic relationship between different VTLs of the two species (Fig. 1a). Based on this phylogeny, the *M. truncatula* genes were assigned as *VTL1–VTL8*. *MtVTL1, MtVTL2* and *MtVTL3* are most similar to *Arabidopsis thaliana VTL1–VTL4*. *MtVTL4*, *MtVTL5*, *MtVTL6* and *MtVTL7* are closely related paralogues on chromosome 4 which are more similar to *Arabidopsis thaliana VTL5* than to other *AtVTL* genes. In contrast, *MtVTL8* has no direct homologue in *Arabidopsis thaliana*, but is closely related to *SEN1* in *L. japonicus* (Hakoyama *et al*., 2011) and *NODULIN‐21* of soybean (*GmNod21*, Delauney *et al*., 1990; renamed *GmVTL1* in Brear *et al*., 2020 and Liu *et al*., 2020). The amino acid sequences of MtVTL8 and LjSEN1 share 75.7% similarity (63.0% identity). Between MtVTL8 and GmNod21 the similarity is 58.7%, and between LjSEN1 and GmNod21 similarity is 63.2%.

Insight into the expression of *M. truncatula VTLs* was primarily obtained from publicly available resources. Three independent studies found that *MtVTL4* transcripts are highly enriched in nodules, compared to low levels in roots and virtually no expression in aerial parts (Benedito *et al*., 2008; Limpens *et al*., 2013; Roux *et al*., 2014). RNA‐seq data (Roux *et al*., 2014; https://iant.toulouse.inra.fr/symbimics) showed that *VTL8* is exclusively expressed in nodules (there is no probe for *VTL8* in the Affymetrix chip used in the other two studies), at levels that are > seven‐fold greater than those of *VTL4* (Fig. 1b). Of the other *MtVTLs*, only *VTL2* is abundantly expressed in nodules, but the levels are similar in roots. Low expression of *VTL1* and *VTL6/7* in nodules are in agreement with Northern blot data for the closest *L. japonicus* homologues, represented by ESTs MWM137c08 and MWM075h10, respectively (Hakoyama *et al*., 2011).

During nodule development, the expression of *MtVTL4* is highest in the infection zone, Zone IId (Roux *et al*., 2014; Fig. 1c) and > four‐fold enriched in infected cells relative to noninfected cells (Limpens *et al*., 2013). *MtVTL8* expression peaks in the Interzone (Zones II–III). This expression pattern is similar to that of *LjSEN1* which increased as the nodule matured (Hakoyama *et al*., 2011). Our own data using the *MtVTL8* promoter for expression of VTL8‐mCherry showed fluorescence signal in infected cells only (see later).

In summary, of the eight *VTL* genes encoded in the *M. truncatula* genome, *VTL8* is the closest homologue of the nodule‐specific *LjSEN1* and *GmNod21* and has a similar expression pattern. In addition, *VTL4* is also specifically induced in nodules, but earlier in the infection process.

### MtVTL4 and MtVTL8 localize to membranes in infected cells

**Fig. 2:**
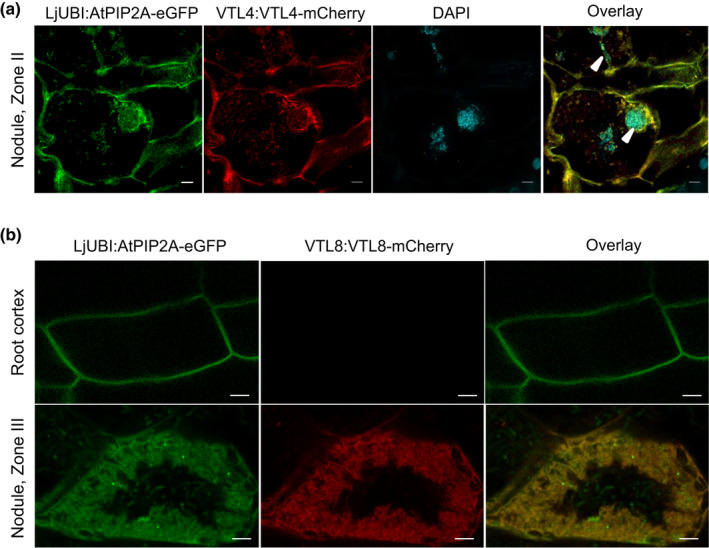
Localization of VTL4 and VTL8 in *Medicago truncatula*. Translational fusions of VTL4 (a) and VTL8 (b) with mCherry fluorescent protein were expressed in roots of wild‐type seedlings, which were nodulated with *Sinorhizobium meliloti* 1021. The *VTL* genes were placed under the control of their own promoters, and co‐expressed with the membrane marker *AtPIP2A‐eGFP* using the constitutive *Lotus japonicus UBIQUITIN* (*UBI*) promoter. (a) Localization of VTL4‐mCherry. The image is of cells in Zone II (infection zone). DAPI staining visualizes DNA including the bacteria in infection threads (arrow heads). Bars, 5 µm. (b) Localization of VTL8‐mCherry. Images of fluorescence signals in the root cortex and Zone III (nitrogen‐fixing zone) are shown. Bars, 5 µm.

To investigate the expression pattern and localization of the VTL4 and VTL8 proteins in nodules, the coding sequences were fused in frame to mCherry using a short C‐terminal linker peptide and expressed under the control of their own promoters in roots of R108 wild‐type seedlings. The presence of a glycine‐rich linker peptide has previously been shown to improve membrane insertion and stability of *Arabidopsis thaliana VTLs* expressed in Baker's yeast (Gollhofer *et al*., 2014). The same vector also contained a constitutively expressed GFP fusion of aquaporin AtPIP2 to visualize membranes (Cutler *et al*., 2002). AtPIP2A has previously been used as a plasma membrane marker in *M. truncatula* (Ivanov & Harrison, 2014) and was shown to internalize to membranes surrounding the infection thread during rhizobium infection (Fournier *et al*., 2008), and to perihyphal membranes and the arbuscule trunk domain of the periarbuscular membrane in mycorrhizal symbiosis (Pumplin & Harrison, 2009). From several membrane markers that we tested, *LjUBIprom:AtPIP2A‐eGFP* was expressed in all nodule cell types and GFP was found in the plasma membrane as well as symbiosomes (Fig. 2), in agreement with reports that the symbiosome membrane contains proteins of plasma membrane origin as well as late endosome markers (Limpens *et al*., 2009).

In roots expressing *VTL4prom:VTL4‐mCherry*, a specific fluorescence signal was found associated with membranes in cells of the infection zone, such as the cell with an infection thread shown in Fig. 2(a). VTL4‐mCherry co‐localized with AtPIP2A‐eGFP to the plasma membrane and was also found in membranes surrounding the infection thread (Fig. 2a, arrow head in merged image).

VTL8‐mCherry was only detected in the characteristically shaped infected cells of the Interzone and not in noninfected neighbouring cells or in root cells (Fig. 2b). This expression pattern is in agreement with RNA‐seq data (Fig. 1b,c), and protein localization studies of GmNod21/GmVTL1 in soybean (Brear *et al*., 2020; Liu *et al*., 2020). While the fluorescence signal was weak, resulting in low‐resolution images, it was clearly confined to the mass of bacteroids surrounding the central vacuole. Co‐localization with AtPIP2A‐eGFP and assuming that both proteins are membrane bound, suggest that VTL8 is localized to the symbiosome membrane. The soybean homologue was indeed found in the enriched symbiosome membrane fraction by proteomics (Brear *et al*., 2020). The weak fluorescence signal of VTL8‐mCherry is surprising given the relatively high transcript levels of endogenous *VTL8*. This may be due to lower transcriptional activity of the domesticated *VTL8* promoter sequence; lack of the native 3′UTR in this construct; or less efficient membrane insertion of the fusion protein leading to protein instability. Although further studies would be beneficial, these results are a first indication that VTL4 and VTL8 localize to intracellular host plant membranes associated with rhizobia.

### *VTL8* is required for nodule development and nitrogen fixation

**Fig. 3:**
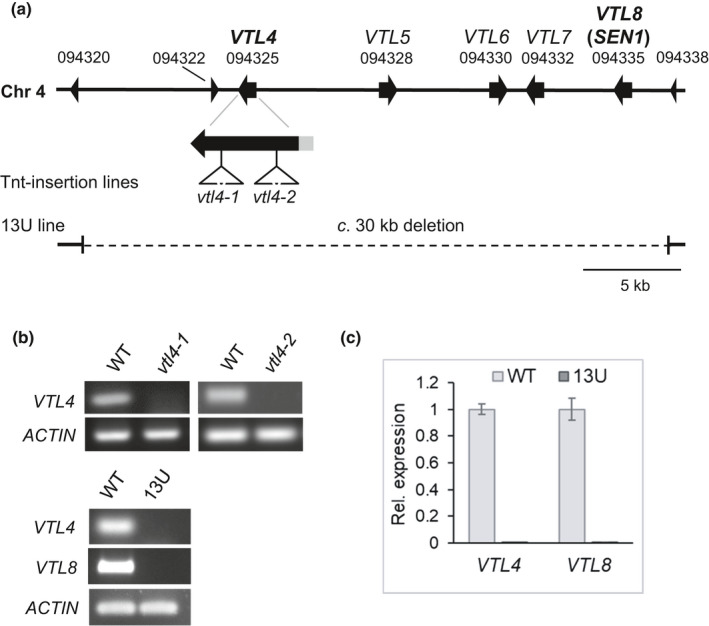
*Medicago truncatula* mutant lines affected in *VTL* gene expression. (a) Arrangement of *VTL* paralogues on chromosome 4. The position of two different *Tnt1* insertions in *VTL4* are indicated by triangles. The deleted segment in the 13U line is indicated by a dashed line. *Medtr4g094320*, *Medtr4g094322* and *Medtr4g094338* encode hypothetical proteins of less than 100 amino acids. (b) Upper panel, RT‐PCR of *VTL4* transcript levels in nodules (28 d post inoculation, dpi) of the parental wild‐type (WT, cultivar R108) and the *vtl4‐1* and *vtl4‐2 Tnt1* insertion lines, NF17463 and NF21016, respectively. Lower pane, RT‐PCR of *VTL4* and *VTL8* transcript levels in nodules of the parental wild‐type (WT, cultivar Jemalong) and the 13U line. (c) Quantitative RT‐PCR results for the expression of *VTL4* and *VTL8* in nodules (28 dpi) of the 13U line relative to the parental wild‐type. The expression was normalized to that of the *UPL7* gene. Error bars represent the SE of the mean from three technical replicates, using cDNA from two different plants for each line.

To study the role of *VTL4* and *VTL8* in nodule development, mutants were obtained from different sources. Two lines with a *Tnt1* insertion in the coding sequence of *VTL4* were identified in the Noble Foundation collection (Fig. 3a), but none were found for *VTL8* despite extensive screening by PCR. However, a mutant line lacking several *VTL* genes was isolated from a collection of Fix‐mutants generated by fast neutron radiation. The rough map position of the deletion in the 13U mutant had been identified previously (Domonkos *et al*., 2013). Microarray hybridization using the genomic DNA of 13U identified a probe set corresponding to the gene *Medtr4g094325* (*VTL4*). Further analysis by PCR amplifications identified a 30‐kb deletion in the 13U genome spanning *VTL4* to *VTL8* (Fig. 3a). Gene expression analysis confirmed the absence of *VTL4* transcript in the *vtl4* mutants and the absence of both *VTL4* and *VTL8* expression in the 13U line (Fig. 3b,c).

**Fig. 4:**
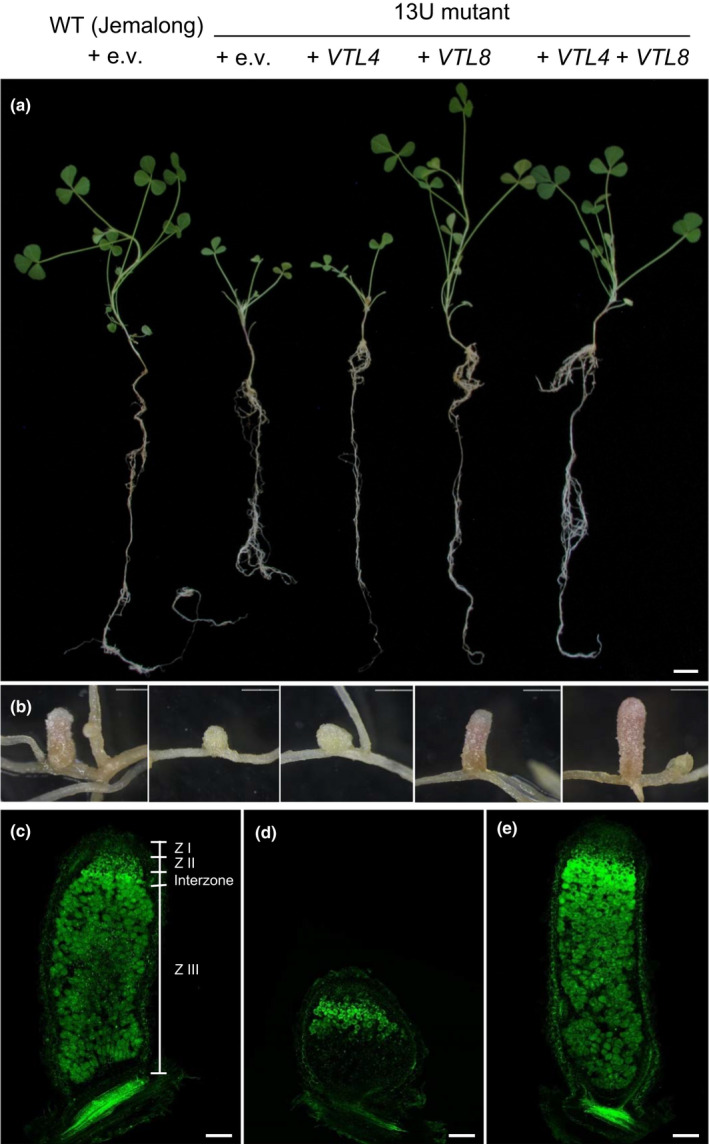
*VTL8* is required for development of functional nodules. (a) Wild‐type *Medicago truncatula* plants (WT, Jemalong) and the 13U mutant transformed with *VTL4*, *VTL8*, *VTL4 + VTL8* or empty vector (e.v.), as indicated. Roots were inoculated with *Sinorhizobium medicae* and images taken 31 d post inoculation (dpi). Bar, 1 cm. (b) Root nodules from plants in (a). Transformed roots were confirmed by DsRed fluorescent protein marker (not shown). Bars, 1 mm. (c) Wild‐type; (d), 13U + e.v. and (e), 13U + *VTL8*, longitudinal sections of nodules stained with SYTO13. Bars, (c–e), 200 μm.

The 13U plants grown in low‐nitrogen substrate were chlorotic and smaller than the parental wild‐type (Fig. 4a), but grew normally upon nitrogen fertilization (Domonkos *et al*., 2013; Fig. S1). The development of 13U nodules was arrested at an early stage and they lacked the typical pink colour of leghaemoglobin (Fig. 4b). Previous studies on 13U nodules showed bacterial colonization of the infection zone and Interzone but only sporadic infection within the nitrogen fixation zone, and no detectable nitrogenase activity (Domonkos *et al*., 2013). To further investigate the defective development of the rhizobia into bacteroids, nodule sections were stained with the nucleic acid binding dye SYTO13. The overall morphology of bacteria in 13U plants appeared similar to wild‐type, but in the interzone they were disorganized rather than orientated toward the vacuoles of infected cells (Fig. S2). Size classification of isolated rhizobia showed that 13U nodules contained mostly poorly elongated bacterial cells, contrasting with a large population of elongated cells between 5 and 9 µm in wild‐type nodules (Fig. S3). The shift to smaller‐sized rhizobial cells is reminiscent of the *dnf7‐2* ineffective mutant identified previously (Horvath *et al*., 2015), suggesting a similar defect in differentiation of the bacteroids in the 13U line.

In order to demonstrate which gene or genes caused the symbiotic phenotype of 13U, genetic complementation experiments were carried out. Transformation with constructs expressing *VTL4* and *VTL8* individually or together showed that expression of *VTL8* alone in the 13U mutant reverted the mutant phenotype to wild‐type. Specifically, vegetative growth of 13U plants + *VTL8* was similar to wild‐type (Fig. 4a), nodules developed fully with the typical pink colour of leghaemoglobin (Fig. 4b), a large Zone III (Fig. 4e) and normal fresh weight per nodule (Fig. S4), indicating that nitrogen fixation was restored. Expression of *VTL4* alone in the 13U mutant had little effect (Fig. 4a,b), although co‐expression with *VTL8* increased nodule fresh weight to above wild‐type values (Fig. S4). Thus, *VTL8* is essential for nodule maturation, whereas *VTL4* has a minor function and *VTL5*, *VTL6* and *VTL7* do not appear to have a function in nodules or in another aspect of plant development.

### *MtVTL4* is required for early bacteroid development

**Fig. 5:**
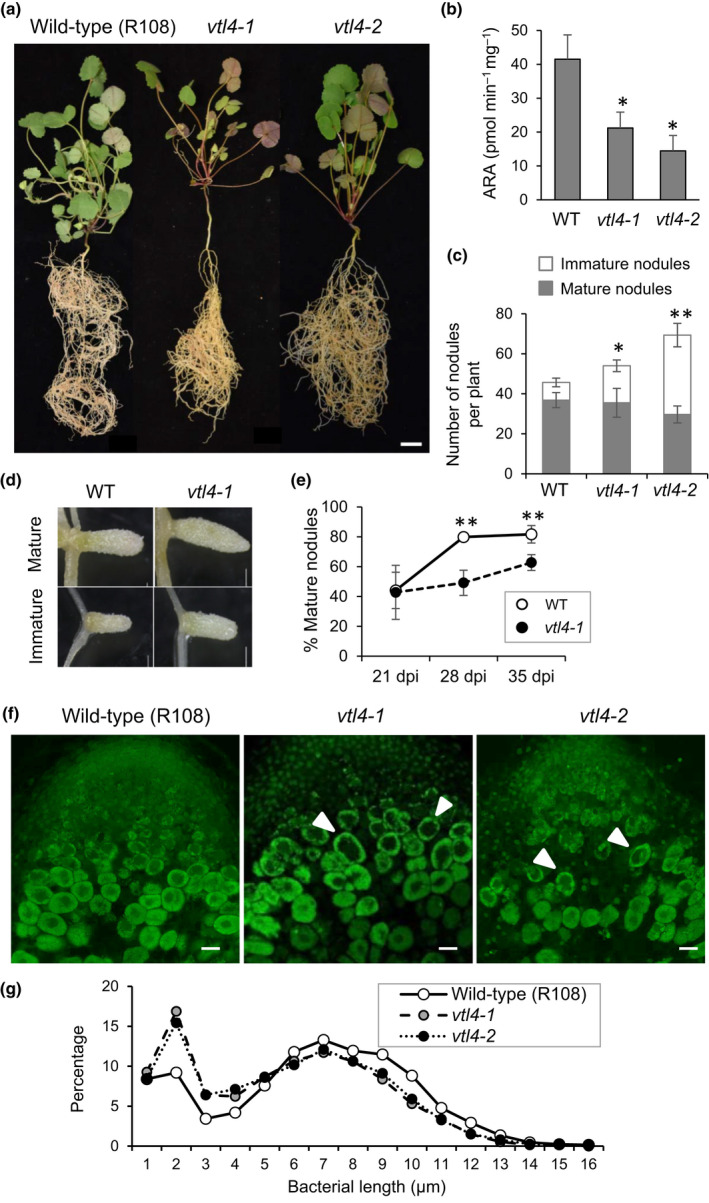
Disruption of *VTL4* delays infection. (a) Wild‐type *Medicago truncatula* (R108) and *vtl4* mutant plants grown under nodulating conditions. The representative images are of 40‐d‐old plants, 35 d after inoculation with *Sinorhizobium meliloti* 1021. Bar, 1 cm. (b) Acetylene reduction activity (ARA), per milligram nodule fresh weight, of the indicated plant lines at 28 d post inoculation (dpi). Values represent the mean ± SE of three measurements, each on two pooled root systems per vial. *, *P* < 0.1, Student's *t*‐test. (c) Number of mature and immature nodules in *vtl4* mutants relative to wild‐type, at 35 dpi. Values represent the mean ± SE of six plants: *, *P* < 0.1; **, P < 0.01 (number of white nodules), Student’s *t*‐test. (d) Representative images of mature and immature nodules in *vtl4‐1* and wild‐type. Bars, 1 mm. (e) Percentage mature nodules per plant at 21, 28 and 35 dpi in wild‐type (open circles) and the *vtl4‐1* mutant (closed circles). Error bars represent SE, *, *P* < 0.1; **, P < 0.01 (*n* = 5–15 plants), Student's *t*‐test. (f) Longitudinal sections of nodules stained with SYTO13, showing part of the infection zone (Zone II). Arrow heads point to infected cells with a larger vacuole and smaller bacteroids. Bars, 100 µm. (g) The distribution of bacteroid cell sizes in wild‐type nodules (WT, R108 represented by open circles) and *vtl4* nodules (grey and black closed circles). The lengths of at least 2500 bacterial cells were measured and their relative distribution in size classes is presented.

To further investigate whether *VTL4* has a role in nodule development, the two *vtl4* mutant lines and wild‐type (R108) were grown on low‐nitrogen substrate and inoculated with rhizobia. While there were no major defects in vegetative development (Fig. 5a), fresh weight measurements showed that *vtl4‐1 and vtl4‐2* plants had significantly less biomass than wild‐type under both low‐ and high‐nitrogen growth conditions (Fig. S1). The nitrogen fixation capacity, measured as acetylene reduction activity in excised roots, showed a *c*. 50% decrease in the *vtl4* mutant lines compared to wild‐type at 28 d post inoculation (dpi) (Fig. 5b). These data indicate that the impaired growth of the two *vtl4* mutant lines may only be partially related to lower nitrogen fixation rates, and it should be noted at this point that the mutant lines were not backcrossed to remove other *Tnt1* insertions (Table S1).

The roots of *vtl4* plants were nodulated, but they had a significantly higher number of immature nodules (lacking leghemoglobin) (Fig. 5c). The total number of nodules per plant also tended to be higher in the mutants, but only significantly so for the *vtl4‐2* data set. There were no macroscopic differences in nodules of *vtl4* mutants and wild‐type (Fig. 5d), suggesting that the higher proportion of immature nodules is due to a delay in development and/or increased initiation of nodule primordia. We followed the percentage of mature nodules over time in the *vtl4‐1* mutant, harvesting plants at weekly intervals. At 21 dpi, the percentage of mature nodules was close to 40% in both wild‐type and *vtl4‐1* plants. At 28 dpi, wild‐type plants had 80% mature nodules, whereas the *vtl4‐1* mutant had only *c*. 50%, increasing to 63% mature nodules at 35 dpi (Fig. 5e).

Cross‐sections stained with SYTO13 showed that the infection of plant cells and bacteroid development proceeded normally in the *vtl4* mutants, except for a subtly different morphology of the infected cells in Zone II (Fig. 5f) where *VTL4* expression reaches its highest levels (Fig. 1c). The early‐stage infected cells appeared ‘hollow’, with a larger vacuole and smaller bacteroids (Fig. 5f, white arrow heads). Measurements of isolated rhizobia showed that *vtl4* mutants have a higher proportion of shorter bacterial cells than wild‐type (Fig. 5g). While this could simply be due to the higher ratio of immature nodules, the decreased nitrogenase activity supports the idea that bacterial development is impaired in the two *vtl4* mutants.

### VTL4 and VTL8 mediate iron transport in yeast

**Fig. 6:**
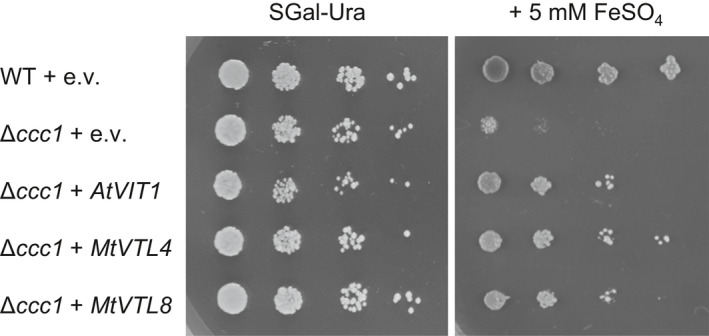
Expression of *VTL4* or *VTL8* restores vacuolar iron transport in Δ*ccc1* yeast. Yeast transformed with pYES2 empty vector (e.v.) or with the indicated *Medicago truncatula VTL* genes were grown on minimal synthetic medium with galactose (SGal) lacking uracil (‐Ura) in a five‐fold serial dilution. Addition of 5 mM iron sulphate to the same medium selects for strains able to transport iron across the vacuolar membrane.

LjSEN1 and its homologues have been suggested to transport iron out of the infected plant cell into the peribacteroid space, based on sequence homology between VTLs and VIT proteins (Hakoyama *et al*., 2011; Brear *et al*., 2013; González‐Guerrero *et al*., 2016). To confirm that the VTL4 and VTL8 proteins are able to transport iron, the genes were expressed in yeast for functional complementation assays. The *VTL* genes were cloned into the pYES2 plasmid under the control of a galactose‐inducible promoter. The plasmids were transformed into a Δ*ccc1* yeast mutant which lacks the vacuolar iron transporter Ccc1 (Li *et al*., 2001). In this mutant, the inability to store iron in the vacuole leads to a severe growth defect in the presence of excess iron in the medium (Fig. 6). Growth can be restored by expressing another vacuolar iron transporter such as Arabidopsis *VIT1* (Kim *et al*., 2006), used here as a positive control alongside the wild‐type strain (Fig. 6). *VTL4* as well as *VTL8* were able to rescue growth of the Δ*ccc1* strain on medium with 5 mM FeSO_4_. Functional complementation was only seen using Δ*ccc1* in the DY150 (W303 derivative) genetic background, but not in the BY4741 strain. This may be due to differences in salt tolerance between the two strains causing an indirect effect on iron homeostasis (Petrezselyova *et al*., 2010). Nevertheless, the results show that MtVTL4 and MtVTL8 are able to transport iron out of the cytosol, either across the vacuolar membrane or plasma membrane.

### Development of a transcriptionally regulated iron reporter in bacteroids

To provide evidence for iron transport *in planta*, we sought to develop an iron‐sensing reporter in symbiotic rhizobium bacteria. Recently, Pini *et al*. (2017) demonstrated the use of transcriptional *lux* reporters for spatio‐temporal mapping of organic molecules in the pea‐rhizobium interaction. The same approach was followed here, but using an iron‐inducible promoter. While iron regulatory mechanisms in free‐living rhizobia are well characterized (Rudolph *et al*., 2006; Todd *et al*., 2006; O’Brian, 2015), much less is known in symbiotic rhizobia. Expression data indicate that most genes involved in iron homeostasis in free‐living rhizobia, for example those for biosynthesis of the siderophore rhizobactin (*rhb*) or for haem‐iron uptake (*hmu*), are not expressed during nodule development (Supporting Information Table S3; Roux *et al*., 2014). However, one gene that is strongly induced is *mbfA* (membrane‐bound ferritin A), with percentage‐wise the highest transcript levels in the proximal Zone II and Interzone of *M. truncatula* nodules, whereas expression is very low in non‐differentiated bacteria of Zone IId. MbfA has been characterized as an iron exporter in *Bradyrhizobium japonicum* and *Agrobacterium tumefaciens* (Bhubhanil *et al*., 2014; Sankari & O’Brian, 2014) and its expression was shown to be regulated by iron in *B. japonicum* (Rudolph *et al*., 2006).

**Fig. 7:**
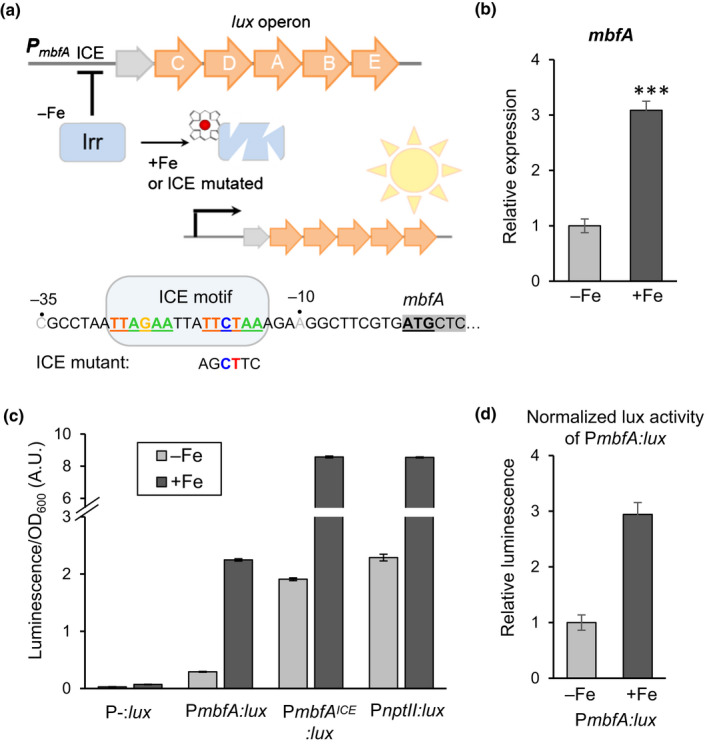
Development of an iron reporter for symbiotic rhizobium bacteria. (a) Diagram of the bacterial P*mbfA:lux* reporter and regulation of its expression by the iron response regulator (Irr) which binds the upstream Iron Control Element (ICE). In iron‐replete conditions, Irr binds iron in the form of haem and is degraded, leading to de‐repression (activation) of target gene expression. (b) Expression of the endogenous *mbfA* gene in free‐living *Sinorhizobium meliloti* 1021 in response to iron. Bacteria were grown in UMS medium without (−Fe) or with 40 µM iron sulphate (+Fe) until mid‐log phase, then harvested for RNA extraction. *mbfA* transcript levels were assayed by RT‐qPCR. Values were normalized to the housekeeping gene *gapA,* and are the mean ± SE of three biological replicates (***, P = 0.0001, Bio‐Rad CFX Maestro software). (c) *Sinorhizobium meliloti* 1021 carrying the *lux* plasmid without promoter (P‐:*lux*); the P*mbfA:lux* reporter; P*mbfA:lux* with a mutated ICE motif; or a constitutive lux reporter with the *nptII* promoter were grown as in (b). Luminescence was measured in a plate reader and corrected for cell density (OD_600_). Values are the mean ± SE of three biological replicates (cell cultures grown in parallel). A.U., arbitrary units. (d) P*mbfA:lux* activity normalized for constitutive lux activity of the P*nptII:lux* reporter in response to iron. Error bars represent SE.

We cloned the promoter region of *S. meliloti mbfA*, including part of an upstream gene and *c*. 100 nucleotides into the coding sequence, and placed it upstream of the *lux* operon on a plasmid, called P*mbfA:lux* (Fig. 7a). To confirm that *S. meliloti mbfA*, and thus P*mbfA:lux*, are regulated by iron, bacteria were grown in iron‐limited and iron‐replete medium to mid‐log phase. Transcript levels of the endogenous *mbfA* gene were analysed by quantitative reverse transcription polymerase chain reaction (RT‐qPCR), and found to be three‐fold induced in the presence of iron (Fig. 7b). Luminescence was measured in bacteria grown under the same conditions but transferred to a plate reader with luminescence detection. *Sinorhizobium meliloti* carrying a promotor‐less *lux* plasmid was used to establish the background signal (Fig. 7c). In the absence of iron, *S. meliloti* carrying the P*mbfA:lux* plasmid gave a luminescence reading that was only slightly above background, showing that *mbfA* promoter activity was strongly repressed. In iron‐replete medium, luminescence was more than seven‐fold increased. Similar results were obtained for early log‐phase and late log‐phase cell cultures (not shown).

Scrutiny of the *S. meliloti mbfA* promoter sequence revealed an ICE motif immediately upstream of the ATG start codon, overlapping with the −35 and −10 elements of the core promoter (Fig. 7a). The ICE motif is the *cis*‐acting element for the iron response regulator Irr in *B. japonicum* (Rudolph *et al*., 2006) and *R. leguminosarum* (Todd *et al*., 2006). The stability of Irr is controlled by haem binding and its regulatory activity is thus highly specific for iron (Yang *et al*., 2005). To show that the iron‐induced P*mbfA:lux* activity is regulated by the ICE motif and Irr, we mutated four nucleotides of the ICE motif with the aim of preventing Irr binding (Fig. 7a). When bacteria were grown in iron‐deficient medium, the ICE mutant of P*mbfA:lux* gave a luminescence signal similar to that of wild‐type P*mbfA:lux* plus iron. Thus, binding of the Irr repressor is abolished in this mutant, without affecting maximal transcriptional activity of the promoter. To further confirm that P*mbfA:lux* activity was regulated by Irr, the P*mbfa:lux* plasmid was transferred into *irrA* ^‐^ mutant and wild‐type strains of *R. leguminosarum* (Wexler *et al*., 2003). In the *irrA* ^‐^ mutant, P*mbfA:lux* was not repressed in low‐iron medium, with a luminescence output similar to a constitutive *lux* reporter construct (Fig. S5a,b).

We noted that the ICE mutant of P*mbfa:lux* has *c*. four‐fold more luminescence in medium with iron, and thus seems to be induced by iron despite the mutation. However, the same effect was observed for a construct with constitutive *lux* expression, P*nptII:lux*, containing the neomycin phosphotransferase II promoter (Frederix *et al*., 2014). Luciferase activity is highly dependent on ATP and FAD, which are more available in healthy cells grown in iron‐replete medium. Lack of iron in the medium strongly limits bacterial growth (Fig. S5c), because iron is an essential element for respiration and many other metabolic processes, affecting the energy status of the cell. When the change in luminescence signal resulting from P*mbfA:lux* expression in response to iron is normalized for luminescence in P*nptII:lux*, the calculated three‐fold increase matches the three‐fold increase in *mbfA* transcript abundance (Fig. 7d).

In summary, tests in free‐living rhizobia show that the P*mbfA:lux* reporter is almost fully repressed in the absence of iron and that, in the presence of iron, the luminescence signal is induced to levels that are comparable to constitutive *lux* expression. Moreover, expression of P*mbfA:lux* is regulated by the *cis*‐acting ICE motif and *trans*‐acting Irr transcription factor, which respond specifically to iron.

### Host plant VTLs are required for iron delivery to the bacteroids

**Fig. 8:**
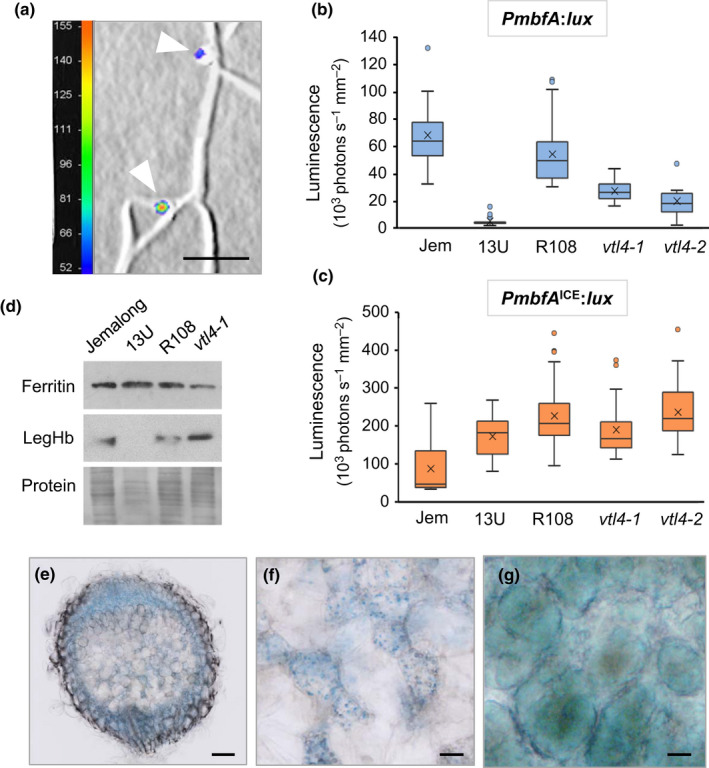
Symbiotic bacteria are iron deficient in nodules lacking VTL4 and VTL8 despite normal iron levels in plant tissues. (a) Detail of an *Medicago truncatula* R108 root inoculated with *Sinorhizobium meliloti* 1021 expressing *PmbfA*:*lux* at 21 d post inoculation (dpi). Luminescence is represented by a colour scale in photons per second, superimposed on a grey‐scale image. The white arrow heads point to nodules. Bars, 5 mm. (b, c) Expression of the bacterial iron reporter *PmbfA:lux* (b) and the deregulated Iron Control Element (ICE) mutant (c) in root nodules at 21 dpi, measured as luminescence with a NightOWL camera. *Medicago truncatula* Jemalong (Jem) was used as wild‐type for the 13U mutant, and *M. truncatula* R108 as wild‐type for the *vtl4* mutants. The data are presented in a box plot, with the box marking the upper and lower quartiles, the middle line represents the median and the symbol × indicates the mean. The whiskers show the spread of the data within the 1.5‐fold interquartile range (*n* = 9 plants for *vtl4‐2*, and *n* ≥ 13 for other lines). (d) Protein blot analysis of ferritin and leghaemoglobin (LegHb) in nodules (21 dpi) of the indicated genotypes. Ponceau S staining of the blot was used as protein loading control (lower panel). (e–g) Histological iron staining of 28‐dpi nodules from (e) 13U mutant plant, (f) enlarged image of the senesced Zone III in 13U, and (g) infected cells in Zone III of wild‐type Jemalong, see Supporting Information Fig. S6 for an image of a whole nodule. Haem‐iron in (g) has a different hue from non‐haem iron. Bars, 0.1 mm (e) and 20 µm (f, g).

To assess the iron status of bacteroids in root nodules, *M. truncatula* seedlings were inoculated with bacteria expressing the P*mbfA*:*lux* reporter, and luminescence was detected with a NightOWL imaging system. Inoculated seedlings grown on vertical agar plates showed no luminescence until the initiation of nodules, similar to the transcriptional *lux* reporters responding to sucrose and gamma‐aminobutyric acid in pea (Pini *et al*., 2017). Luminescence was associated with the central part of the nodule (Fig. 8a), correlating with published RNA‐seq data for *mbfA* expression (Table S3).

To investigate whether mutations in *VTL4* and *VTL8* affect the amount of iron perceived by endosymbiotic bacteria, the mutant lines (13U, *vtl4‐1* and *vtl4‐2*) and their relevant wild‐types (R108 and A17) were grown on Terragreen–sand to obtain a well‐developed root system. *Sinorhizobium meliloti* 1021 was used to inoculate all plant genotypes. While less effective in nodulating *M. truncatula* Jemalong, the parent line of the 13U mutant, the number of nodules were sufficient for quantitative analysis. At 21 dpi, plants were dug up, washed and the roots were imaged for luminescence. The values (in photons per second, normalized to area) are presented as a box plot, a statistical graph method that summarizes the whole data set. The 13U mutant nodules showed 90% decrease in luminescence, and *vtl4* mutant nodules had a reduction in luminescence signal of 50%, compared to their respective wild‐types (Fig. 8b). Because the lower P*mbfA:lux* signal could be due to fewer or ATP‐depleted bacteria in the mutant nodules, we also analysed luminescence in roots inoculated with the constitutive ICE mutant reporter, P*mbfA^ICE^:lux*. As with the wild‐type reporter, luminescence signal was restricted to the nodules, but the area‐normalized values were similar in the *vtl4* mutants and R108 parental line. In the 13U line, luminescence from the P*mbfA^ICE^:lux* reporter was slightly increased compared to the control line (Fig. 8c). Thus, the number or total mass of energy‐replete bacteria in wild‐type and mutant nodules are comparable, regardless of differences in development, but the lower P*mbfA:lux* signal indicates that the bacteria in *vtl4* and 13U mutants are iron deficient.

To investigate to what extent iron homeostasis in the plant is altered in the 13U and *vtl4* mutants, which could indirectly affect the iron status of the bacteroids, protein extracts of nodules were analysed for ferritin. The expression of this iron storage protein is strongly correlated with intracellular iron levels (Briat *et al*., 2010). The ferritin levels in 13U and *vtl4* nodules were similar in their respective wild‐type controls, despite a complete lack of leghaemoglobin in 13U nodules (Fig. 8d). In agreement with these findings, the total iron content was 1.05 ± 0.05 mg g^−1^ dry weight for wild‐type nodules and 1.14 ± 0.14 mg g^−1^ for 13U nodules. Interestingly, staining of nonhaem iron in 13U nodules using Perls’ reagent showed a mosaic of cells with and without iron in the senescing region which corresponds to the nitrogen fixation zone, Zone III (Fig. 8e). At higher magnification, the iron appears associated with small organelles (Fig. 8f). In wild‐type nodules, the infected cells in Zone III contain a high concentration of haem‐iron which stains a greenish‐blue, whereas uninfected cells have little iron staining (Figs 8g, S6).

In summary, these data indicate that VTL4 and VTL8 are specifically required for iron delivery to the bacteroids, but not for transport of iron into host plant cells.

## Discussion

Because of a high demand for metals, nodules provide an interesting system to study the function of transporters and other metal homeostasis genes in a compartmentalized plant cell. After iron is imported across the plasma membrane into an infected cell, it is distributed between the mitochondria, plastids and the differentiating bacteroids. Our functional characterization of two *VTL* genes that are specifically expressed in root nodules of *M. truncatula* strongly indicates they are required for iron delivery to the bacteroids. Evidence for iron as the substrate of VTL4 and VTL8 is based on (1) homology with the vacuolar iron transporter VIT; (2) yeast complementation studies, and (3) the use of a bacterial iron reporter. VIT proteins function as proton‐dependent antiporters of Fe^2+^ and other divalent ions including Mn^2+^ and Co^2+^, although the transport of iron is clearly the main physiological function (e.g. Kim *et al*., 2006). VTLs lack the cytosolic loop proposed to confer substrate specificity (Kato *et al*., 2019), however, the ability of VTLs to partially restore growth of yeast defective in vacuolar iron transport, indicate that iron is indeed a substrate. Nevertheless, transport of other metals such as Mn^2+^ should be investigated, for example using yeast complementation (*pmr1* mutant), Xenopus oocytes studies, or *in vitro* transport studies with reconstituted membrane vesicles.

The third piece of evidence for iron transport is that signal output of a bacterial iron reporter was decreased in the plant *vtl* mutants. Because the transcriptional *lux* reporter was newly developed for this study, we will discuss the pros and cons here.

Specificity and sensitivity are key parameters for any reporter or biosensor. Building on published knowledge regarding iron regulation in rhizobia and other alpha‐proteobacteria (O’Brian, 2015), we showed that the *S. meliloti mbfA* promoter used for the transcriptional reporter is repressed by the Irr transcription factor binding to the ICE motif. Haem‐dependent control of Irr stability makes this regulatory mechanism highly specific for iron. Irr responds to micromolar iron concentrations in the medium (Yang *et al*., 2005), however in our assays we used 40 µM iron, to make sure the bacterial culture would remain iron‐replete during growth of the culture and sequestration of iron into biomass. The iron concentration of the medium is likely to be directly correlated with the intracellular ‘free’ iron concentration, by means of specific iron transporters, which in turn is correlated with haem biosynthesis and regulation by Irr. However, it is technically challenging to obtain a precise figure for intracellular ‘free’ iron or haem, which are kept within a narrow range by homeostatic control. Thus, the reporter cannot be used to measure the extracellular or intracellular iron concentration, but merely shows whether the cells are iron‐limited or iron‐replete (the ‘iron status’ of the cell).

Luciferase activity was chosen as a reporter because it can be used *in vivo* with light‐sensitive CCD cameras (Pini *et al*., 2017), has a greater dynamic range and better signal‐to‐noise ratio than GFP. In particular the virtually zero background signal in plant tissue and in the repressed state of the *mbfA* promoter, are critical. The resolution of CCD cameras is unfortunately low (in millimetres), although systems with 0.1 mm resolution are now available. A drawback of luciferase is that it is dependent on ATP and thus the energy status of the cell.

We tested the reporter in free‐living bacteria, with the caveat that the results are not necessarily transferrable to bacteroids. The latter are much larger in size and do no longer divide. However, expression of *mbfA* and *irr* in bacteroids (Table S3) suggest that Irr operates in both free‐living and symbiotic bacteria. Another potential issue with the reporter system was that the 13U mutant does not have fully developed bacteroids, and the nodules may not have many live bacteria at all. This was refuted using the ICE mutant construct, which showed sufficient luminescence signal in 13U nodules (and thus bacteria that were not ATP‐limited), compared to low luminescence of the wild‐type reporter. This allowed us to conclude that the strongly decreased luminescence signal is due to repression of *mbfA:lux* transcription and thus to iron deficiency.

Expression patterns and our mutant studies showed that the two nodule‐specific *VTL* genes in *M. truncatula* are involved in iron transport at different times of the infection process. This may reflect the findings of older studies in peanut and lupin, that iron is required for both nodule initiation and further development (O’Hara *et al*., 1988; Tang *et al*., 1990). Phenotypic observations of the *vtl4* and 13U mutants are in agreement with the differential expression patterns of *VTL4* and *VTL8* in nodules. VTL4 is thought to provide iron to the bacteria when they are dividing in the infection thread. Lack of VTL4 results in less elongated bacteroids and more immature nodules, either because nodule development is delayed or because more nodules are initiated. These phenotypes could be due to sub‐optimal iron delivery, analogous to the compromised growth of iron‐limited free‐living bacteria (Fig. S3c). The minor effects of knocking out *VTL4* on nodule and bacteroid development suggest that VTL4 is not the only source of iron. At this stage, the bacteria may still have some iron stored from before infection, or simply need little iron. In contrast, VTL8 is critical for full differentiation of the bacteroids into nitrogen‐fixing symbionts. In line with this, *VTL8* expression peaks just before the induction of the bacterial *nif* genes for nitrogenase when large amounts of iron are needed for incorporation in the iron–sulphur cofactors of the abundant nitrogenase protein (Brear *et al*., 2013). It was previously shown that bacteria unable to express nifD or nifK, two main structural proteins of nitrogenase, differentiate into late‐stage bacteroids, but then undergo early senescence (Hirsch *et al*., 1983).

Interestingly, iron homeostasis in the nodules is hardly affected in 13U mutants. For instance, no accumulation of iron was observed in the vascular bundles or other parts, unlike the *LjMATE* mutants (Takanashi *et al*., 2013; Kryvoruchko *et al*., 2018). Iron‐rich bodies were observed in the equivalent of Zone III, possibly proplastids unable to use iron for haem biosynthesis, or degenerated symbiosomes before iron is recycled. Thus, the cells in Zone III of the 13U mutant receive sufficient iron, and providing more iron to the plant is unlikely to bypass the block in iron delivery to the bacteroids. In Arabidopsis, iron accumulation is suppressed by BRUTUS, an iron‐regulated E3 ligase (Rodriguez‐Celma *et al*. 2019; Selote *et al*., 2015). The *L. japonicus* homologue of BRUTUS was shown to play an important role in nodule development (Shimomura *et al*., 2006), although iron homeostasis in the *Lj brutus* mutant remains to be investigated.

Phylogenetic analysis shows that *VTL8*, *LjSEN1* and *GmNOD21* form a separate clade of *VTL* genes. It is therefore likely that the gene was recruited specifically for symbiosis in the ancestral legume species. Why was a *VIT‐*like gene, but not *VIT*, co‐opted for this function? Perhaps VIT could not easily be recruited to the symbiosome membrane during bacteroid development as it is specifically localized to the vacuole membrane, and symbiosomes do not acquire vacuole marker proteins until shortly before senescence (Limpens *et al*., 2009). Alternatively, it is possible that VTLs transport both Fe^2+^ and Fe^3+^, or Fe^3+^ exclusively, because the cytosolic loop is missing in VTLs. Interestingly, VTLs have a high degree of sequence similarity to the transmembrane domain of MbfA, which also lacks the cytosolic loop but has an additional N‐terminal ferritin‐like domain (Bhubhanil *et al*., 2014; Sankari & O’Brian, 2014). It has been suggested that this domain oxidizes Fe^2+^ to Fe^3+^, which is then transported by the membrane domain. It would be interesting to see if plant ferritin, which accumulates transiently in Zone II, is able to associate with VTL8 to deliver iron to the peribacteroid space.

It is still not clear how iron is taken up by the bacteroids. As noted earlier, iron uptake genes that are active in free‐living rhizobia (Johnston *et al*., 2001) are generally not induced in the symbiont stage (Table S3, Roux *et al*., 2014, and reviewed in Abreu *et al*. (2019)). The Fe^2+^ transporter FeoAB plays a role in iron transport in *Bradyrhizobium* and *feoA* or *feoB* deletion strains produced ineffective nodules on soybean (Sankari & O’Brian, 2016). However, there are no *feoAB* genes in the *S. meliloti* genome. Alternatively, iron may be taken up as Fe^3+^‐citrate or Fe^3+^‐malate via tricarboxylic acid transporters, which are highly induced in expression. In any case, solubility of iron in the peribacteroid space should not be an issue for efficient iron uptake, because of acidification of the peribacteroid space towards the onset of nitrogen fixation (Pierre *et al*., 2013). The iron‐regulated *lux* reporter developed for our study should help to characterize the bacterial iron transporters in nodules. It is important to note that the expression of *MbfA* indicates that bacteroids are saturated with iron, and need to export it back to the peribacteroid space to protect themselves from oxidative stress. Thus, low‐affinity uptake systems may be sufficient for the iron requirements of bacteroids.

In summary, this study provides confirmation that VTL8/SEN1 is the main iron transporter across the symbiosome membrane, but it also opens up new questions regarding iron homeostasis in nodules. In addition to identifying the iron species that is exported by the infected plant cell (and by the bacteria through mbfA), key questions are how iron is delivered to VTL8/SEN1 and how it is partitioned between haem biosynthesis for leghaemoglobin and export to the bacteroids. Additionally, the role of VTLs in iron homeostasis in plants in general needs to be further addressed.

## Author contributions

JHW, RTG, GK‐K, AD, BH, EMB and MF performed the research and helped with designing experiments and data analysis; PK and JB analysed and interpreted the data and wrote the manuscript. JHW and GK‐K contributed equally to this work.

## Supporting information


**Fig**.** S1** Growth of wild‐type, 13U and *vtl4 Medicago truncatula* with and without nitrogen.
**Fig**.** S2** Cytology of infected cells in the 13U mutant.
**Fig**.** S3** Distribution of bacteroid cell sizes in nodules of wild‐type and the 13U mutant.
**Fig**.** S4** Nodule fresh weight of complemented 13U lines.
**Fig**.** S5** Regulation of *mbfA* expression by IrrA and iron.
**Fig**.** S6** Iron staining of nodules using Perls’ reagent.
**Methods S1** Additional information on Materials and Methods.
**Table S1** *Tnt1* insertions in *Medicago truncatula* lines NF17463 (*vtl4‐1*) and NF21016 (*vtl4‐2*).
**Table S2** Primers and other oligonucleotides.
**Table S3** Expression of *Sinorhizobium meliloti* genes involved in iron homeostasis during nodule development.
Please note: Wiley Blackwell are not responsible for the content or functionality of any Supporting Information supplied by the authors. Any queries (other than missing material) should be directed to the *New Phytologist* Central Office.
