# Supplementary material for: The Medicago truncatula Vacuolar iron Transporter‐Like proteins VTL4 and VTL8 deliver iron to symbiotic bacteria at different stages of the infection process
Source: New Phytol. 2020 Jul 16;228(2):651–66. doi: 10.1111/nph.16735 (PMC7540006; doi:10.1111/nph.16735)
Supplement: Supplementary file 1 — Fig. S1 Growth of wild‐type, 13U and vtl4 Medicago truncatula with and without nitrogen. Fig. S2 Cytology of infected cells in the 13U mutant. Fig. S3 Distribution of bacteroid cell sizes in nodules of wild‐type and the 13U mutant. Fig. S4 Nodule fresh weight of complemented 13U lines. Fig. S5 Regulation of mbfA expression by IrrA and iron. Fig. S6 Iron staining of nodules using Perls’ reagent. Methods S1 Additional information on Materials and Methods. Table S1 Tnt1 insertions in Medicago truncatula lines NF17463 (vtl4‐1) and NF21016 (vtl4‐2). Table S2 Primers and other oligonucleotides. Table S3 Expression of Sinorhizobium meliloti genes involved in iron homeostasis during nodule development. Please note: Wiley Blackwell are not responsible for the content or functionality of any Supporting Information supplied by the authors. Any queries (other than missing material) should be directed to the New Phytologist Central Office. [file NPH-228-651-s001.pdf]

New Phytologist

New Phytologist Supporting Information

Article title: **The _Medicago truncatula_ Vacuolar iron Transporter-Like proteins VTL4 and VTL8 deliver iron to symbiotic bacteria at different stages of the infection process**

Authors: Jennifer H. Walton, Gyöngyi Kontra-Kováts, Robert T. Green, Ágota Domonkos, Beatrix Horváth, Ella M. Brear, Marina Franceschetti, Péter Kaló and Janneke Balk

Article acceptance date: 27 May 2020

The following Supporting Information is available for this article:

Fig. S1 Growth of wild-type, 13U and *vtl4 Medicago truncatula* with and without nitrogen.

Fig. S2 Cytology of infected cells in the 13U mutant.

Fig. S3 Distribution of bacteroid cell sizes in nodules of wild type and the 13U mutant.

Fig. S4 Nodule fresh weight of complemented 13U lines.

Fig. S5 Regulation of *mbfA* expression by IrrA and iron.

Fig. S6 Iron staining of nodules using Perls' reagent.

Table S1 *Tnt1* insertions in *Medicago truncatula* lines NF17463 (*vtl4-1*) and NF21016 (*vtl4-2*).

Table S2 Primers and other oligonucleotides.

Table S3 Expression of *S. meliloti* genes involved in Fe homeostasis during nodule development.

Methods S1 Additional information on Materials and Methods.

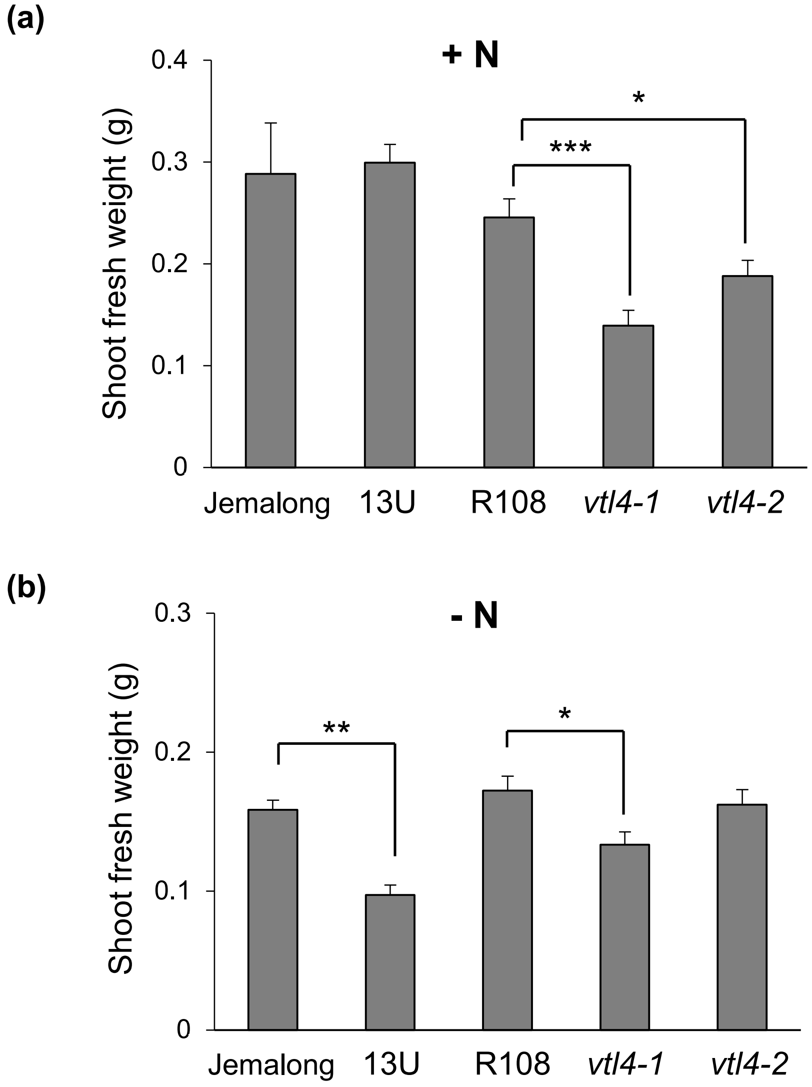

Figure S1. Growth of wild-type, 13U and *vtl4 Medicago truncatula* with and without nitrogen.
(a) Shoot fresh weight of plants grown on zeolite saturated with Gibson liquid medium (Gibson and Nutman, 1960) containing 10 mM NH_4NO_3 and watered with the same once a week for 3 weeks, then harvested at 4 weeks. (b) Shoot fresh weight of plants grown on zeolite and inoculated with *Sinorhizobium medicae* WSM419 (Jemalong, 13U) or *S. meliloti* 1021 (R108, *vtl4-1*, *vtl4-2*), harvested at 4 weeks. Bars represent the mean ± SE. **P*<0.1, ***P*<0.01, ****P*<0.001 Student's *t*-test. (n ≥ 15)

Reference: **Gibson AH, Nutman PS 1960.** Studies on the physiology of nodule formation: with two figures in the text: a reappraisal of the effect of preplanting. *Annals of Botany* **24**: 420–433.

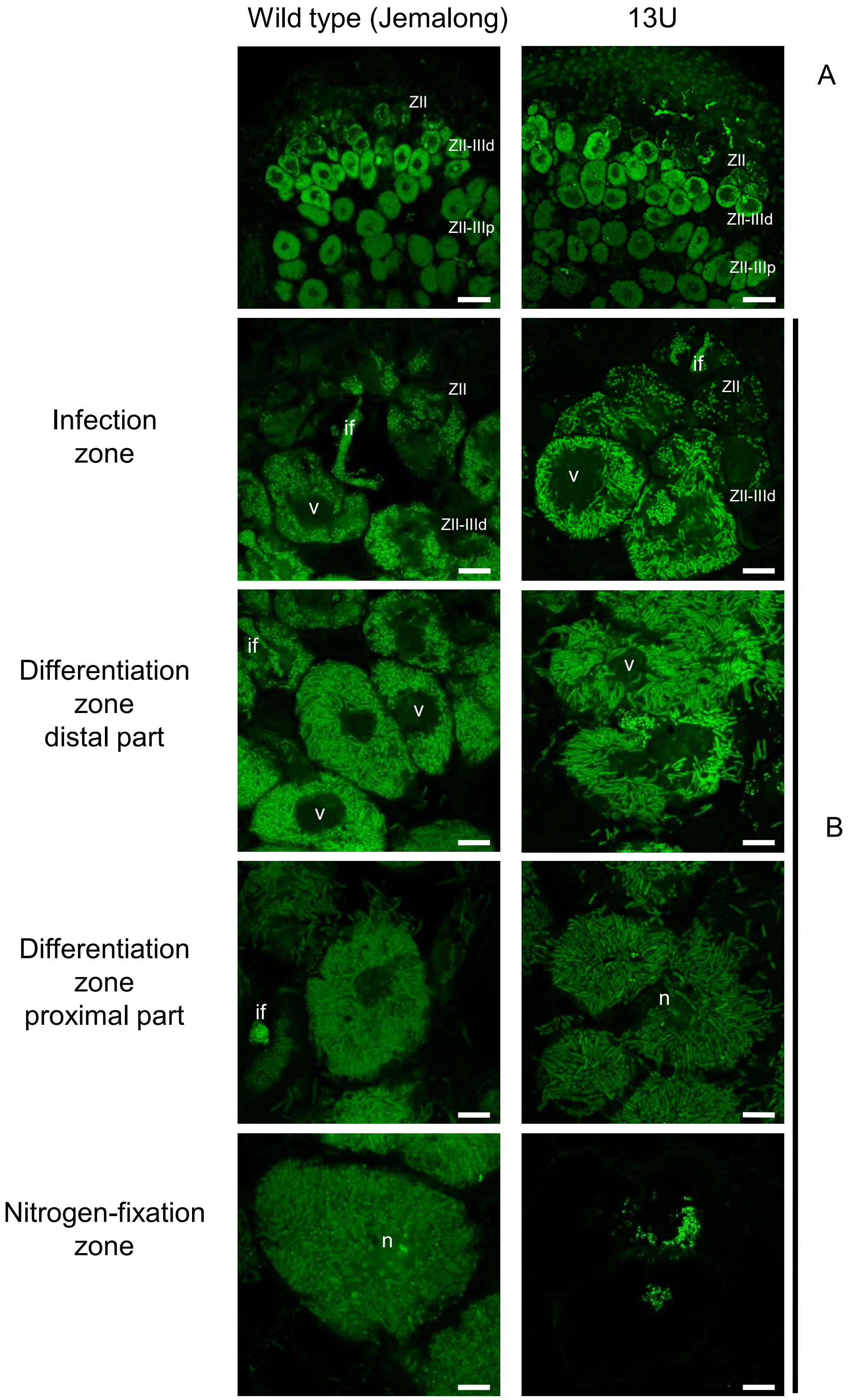

Figure S2. Cytology of infected cells in the 13U mutant.

Confocal images of SYTO13-stained sections of *Medicago truncatula* wild-type (Jemalong) and 13U nodules three weeks post inoculation with *Sinorhizobium medicae* WSM419. 13U nodules show disorganized dispositon of elongated bacteroids in the differentiation zone and have a nitrogen-fixation zone devoid of symbiotic cells. Scale bars (A) 50 µm, (B) 10 µm.

ZII: infection zone, ZII-IIId: differentiation zone distal part, ZII-IIIp: differentiation zone proximal part. if: infection thread, v: vacuole, n: nucleus.

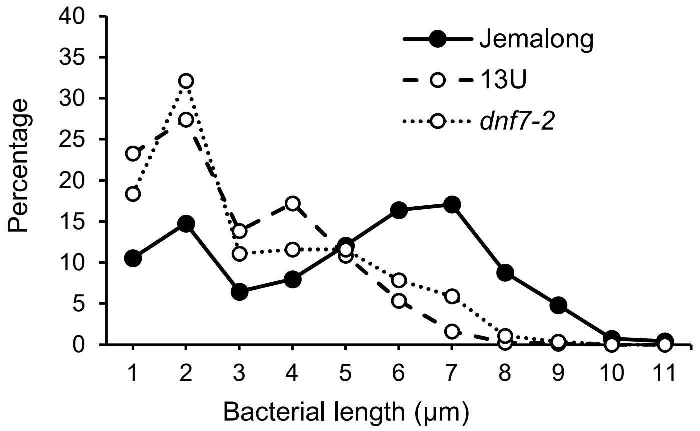

Figure S3. Distribution of bacteroid cell sizes in nodules of wild type and the 13U mutant. The 13U line and respective wild type of *Medicago truncatula* Jemalong were inoculated with *Sinorhizobium medicae* WSM419.The *dnf7-2* mutant defective in terminal bacteroid differentiation was analysed for comparison. Bacteria were isolated from the nodules, stained with propidium iodide, and imaged by confocal laser scanning microscopy. The lengths of at least 800 bacterial cells (wild type Jemalong and 13U) and 400 cells (*dnf7-2*) were measured and their relative distribution in size classes is shown.

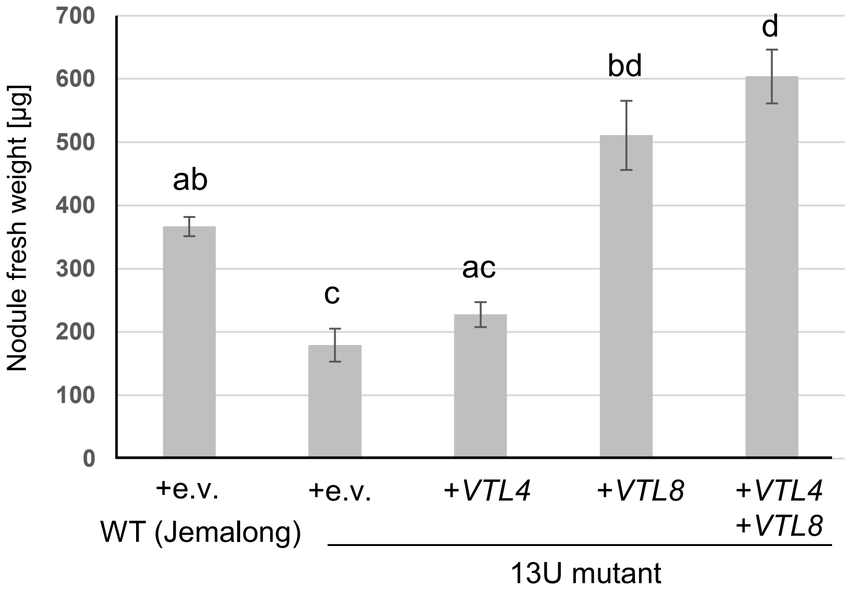

Figure S4. Nodule fresh weight of complemented 13U lines. For each root transformation, nodules were pooled to determine the total fresh weight, divided by the number of nodules. The values are the mean of 3 independent transformations ± SE. Letters shared in common between the transformation indicate no significant difference (one-way ANOVA, Tukey’s HSD tests).

(a)

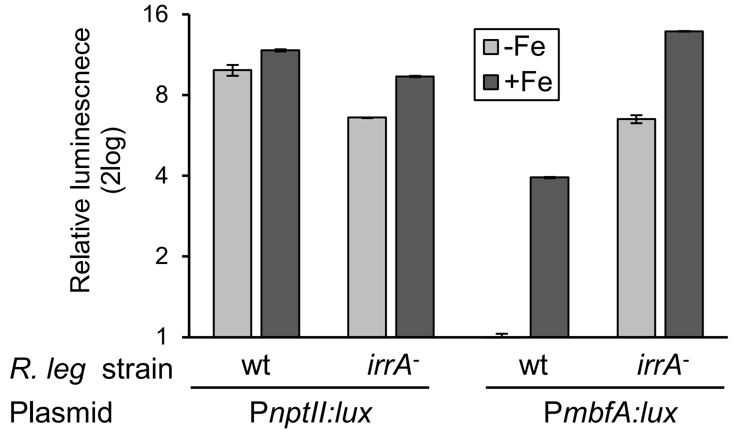

(b) *R. leguminosarum* bv. *viciae*

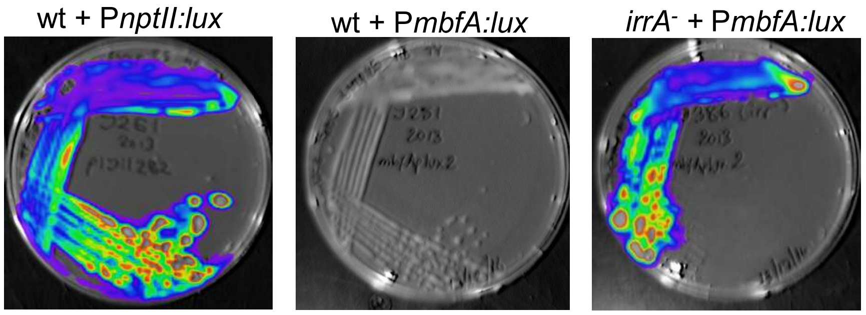

(c) Growth of *S. meliloti* 1021 in response to iron

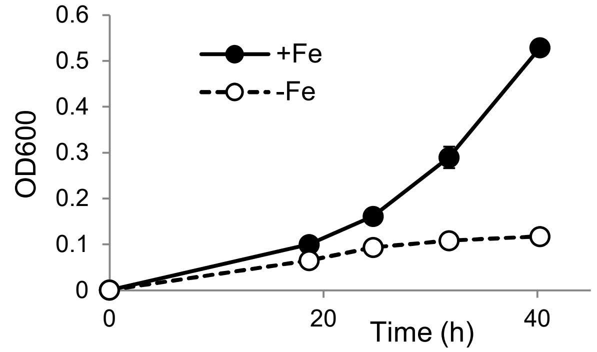

Figure S5. Regulation of *mbfA* expression by IrrA and iron.

(a) Expression of the *Sinorhizobium meliloti mbfA* promoter is regulated by the Iron response regulator IrrA in *Rhizobium leguminosarum.* The P*mbfA:lux* reporter plasmid was transferred by conjugation into a wild-type strain and an *irrA*$^{-}$ mutant strain of *R. leguminosarum* (Wrexler *et al*., 2003; Todd *et al*., 2006). Cells were grown in liquid medium with or without 40 µM $FeSO_4$ (as indicated) and luminescence was measured at an OD600 ~ 1. Values represent the mean ± SE of 3 bacterial cultures.

(b) *R. leguminosarum* strains as in (a), grown on TY agar plates (iron sufficiency). Luminescence was imaged with the NightOWL imaging system, and represented as an artificial colour scale from blue (low) to red (high).

(c) A typical growth curve of *S. meliloti* 1021 in UMS medium with and without iron, as used for studies of the P*mbfA:lux* reporter. Values represent the mean ± SE of 3 bacterial cultures.

References: **Wexler M et al.,** *Microbiology* **149**: 1357–1365.
Todd JD et al., *Molecular Genetics and Genomics* **275**: 564–577.

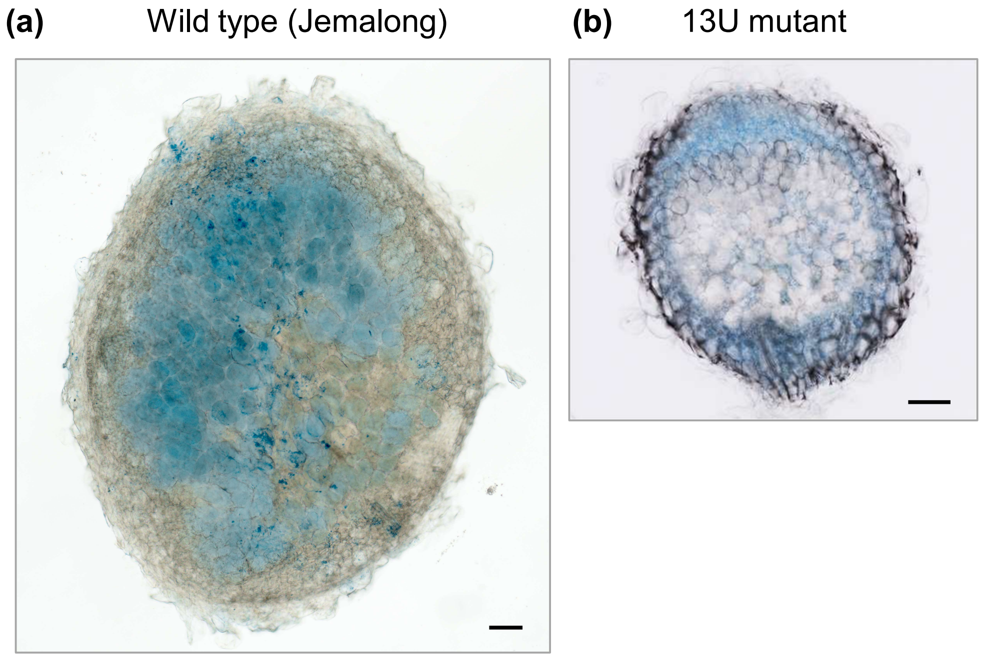

Figure S6. Iron staining of nodules using Perls' reagent.
Medicago truncatula Jemalong nodules at 28 dpi from wild type (a) and 13U mutant plants (b) were stained for iron using potassium iron cyanide which gives a blue colour, also known as Perls' staining. To stain haem-iron in the infected cells of wild-type nodules, the ferricyanide salt was used instead of ferrocyanide, which results in a greenish-blue colour. The images are of 50-100 µm sections made with a vibratome, imaged with a Zeiss AXIO Imager Z2 (left) or Leica DM600 microscope (right). Scale bars are 0.1 mm.

New Phytologist

Table S1 ***Tnt1*** **insertions in *Medicago truncatula* lines NF17463 (*vtl4-1*) and NF21016 (*vtl4-2*)**
Flanking sequence tags (FSTs) of *Tnt1* insertions listed on https://medicago-mutant.noble.org/mutant/tnt1.php (accessed on 10 December 2019).

Line NF17463

FST Sequence ID	Start	End	Target	Nearest open reading frame
NF17463_high_1	33178439	33177673	chr3	**1.291 Kb upstream** of **Medtr3g073590** electron carrier/protein disulfide oxidoreductase<br>Coexpressed with genes in root-specific subnetwork
NF17463_high_2	N/A	N/A	N/A	
NF17463_high_3	9496496	9495891	chr7	**3.022 Kb upstream** of **Medtr7g028190** hypothetical protein
NF17463_high_4	38043260	38042798	chr7	**0.355 Kb upstream** of **Medtr7g095150** ovate transcriptional repressor
NF17463_high_5	55083794	55083370	chr3	**0.875 Kb upstream** of **Medtr3g117625** hypothetical protein<br>**0.588 Kb upstream** of **Medtr3g117630** SAUR-like auxin-responsive family protein
NF17463_high_6	34669508	34669884	chr4	**In coding sequence (32 nt before Stop)** of **Medtr4g088035** LOB domain protein
NF17463_high_7	8484346	8484697	chr2	**2.083 Kb upstream** of **Medtr2g023920** IGR motif protein<br>Coexpressed with genes in roots specific coexpression subnetwork
NF17463_high_8	8483856	8484081	chr2	**2.021 Kb upstream** of **Medtr2g023910** fungal proteinase A, aspartic proteinase superfamily protein; Coexpressed with genes in nodule-specific subnetwork
NF17463_low_1	33178439	33177673	chr3	Same as High_1
NF17463_low_2	N/A	N/A	N/A	
NF17463_low_3	9496496	9495891	chr7	Same as High_3
NF17463_low_4	37733541	37733004	chr4	**In coding sequence** of **Medtr4g094325** vacuolar iron transporter-like protein (**VTL4**)<br>Differentially upregulated in nodules in symbiotic conditions vs. roots
NF17463_low_5	5026917	5027450	chr5	**In 5' UTR** of **Medtr5g014810** hypothetical protein
NF17463_low_6	38043260	38042798	chr7	Same as High_4
NF17463_low_7	55083794	55083370	chr3	Same as High_5
NF17463_low_8	32260156	32259760	chr3	**In 2nd exon** of **Medtr3g071870** cation/H+ exchanger 3
NF17463_low_9	34669508	34669884	chr4	Same as High_6
NF17463_low_10	8484346	8484697	chr2	Same as High_7
NF17463_low_11	8483856	8484081	chr2	Same as High_8

Line NF21016[a]

FST Sequence ID	Start	End	Target	Nearest open reading frame
NF21016_high_1	42395878	42395404	chr8	**In 1st exon or in 1st intron of alternative transcript** of **Medtr8g100065** GRAM domain protein/ ABA-responsive-like protein; Coexpressed with genes in nodule-specific subnetwork
NF21016_high_10	1250651	1250893	chr1	**0.109 Kb upstream** of **Medtr1g009260** hypothetical protein
NF21016_high_11	4367025	4367250	chr6	**9.599 Kb downstream** of **Medtr6g013530** circadian clock coupling factor ZGT
NF21016_high_12	53127876	53127655	chr4	**In 2nd intron** of **Medtr4g127790** lysine-tRNA ligase<br>Coexpressed with genes in nodule-specific subnetwork
NF21016_high_13	38186924	38186837	chr1	**1.138 Kb upstream** of **Medtr1g085500** rhicadhesin receptor

				**0.193 downstream** of **Medtr1g085510** Pre-mRNA-splicing factor SLU7-like protein
NF21016_high_2	8899086	8898661	chr8	**In 5th intron of** of **Medtr8g024260** PRR response regulator<br>Coexpressed with genes in leaf-specific subnetwork
NF21016_high_3	42089104	42089520	chr1	**In 3' UTR** of **Medtr1g093800** Clavata3/ESR (CLE) gene family member MtCLE18<br>Differentially downregulated in nodules in symbiotic conditions vs. roots.
NF21016_high_4	30395194	30395584	chr7	**In 3rd exon** of **Medtr7g080000** WRKY family transcription factor
NF21016_high_5	41260717	41261096	chr2	**In the 5' UTR** of **Medtr2g096630** transmembrane protein, putative
NF21016_high_6	19056192	19056573	chr5	**In last (7th) exon** of **Medtr5g043350** polygalacturonase/glycoside hydrolase family protein
NF21016_high_7	1186129	1185804	chr2	**In 1st intron** of **Medtr2g007890** bidirectional sugar transporter
NF21016_high_8	4055352	4055228	chr3	**2.132 Kb downstream** of **Medtr3g014320** LRR and NB-ARC domain disease resistance protein
NF21016_high_9	28957983	28957779	chr1	**3.526 Kb upstream** of **Medtr1g067220** hypothetical protein<br>Coexpressed with genes in leaf-specific subnetwork<br>**3.391 Kb downstream** of **Medtr1g067230** replication A1-like protein, putative
NF21016_low_1	42395878	42395404	chr8	Same as High_1
NF21016_low_10	28957983	28957779	chr1	Same as High_9
NF21016_low_11	1250651	1250893	chr1	Same as High_10
NF21016_low_12	30101668	30101427	chr4	**0.149 Kb downstream** of **Medtr4g078180** hypothetical protein<br>Differentially downregulated in leaf with ammonium vs. roots with ammonium comparison<br>**0.497 Kb downstream** of **Medtr4g078190** hypothetical protein
NF21016_low_13	4367025	4367250	chr6	Same as High_11
NF21016_low_14	53127876	53127655	chr4	Same as High_12
NF21016_low_15	38186924	38186837	chr1	Same as High_13
NF21016_low_2	37947006	37946688	chr4	**0.223 Kb upstream** of **Medtr4g094415** MAP kinase kinase kinase-like protein<br>Coexpressed with genes in leaf-specific subnetwork
NF21016_low_3	8899086	8898661	chr8	Same as High_2
NF21016_low_4	42089104	42089520	chr1	Same as High_3
NF21016_low_5	30395194	30395584	chr7	Same as High_4
NF21016_low_6	41260717	41261096	chr2	Same as High_5
NF21016_low_7	19056192	19056573	chr5	Same as High_6
NF21016_low_8	1186129	1185804	chr2	Same as High_7
NF21016_low_9	4055352	4055228	chr3	Same as High_8

[a]The *Tnt1* insertion in Medtr4g094325 (VTL4) was identified by PCR screening and is not listed in the *Medicago* mutant database.

Table S2 Primers and other oligonucleotides

Oligonucleotide	Purpose	Sequence (5'-3')
	Genotyping of Tnt lines	TCCTTGTTGGATTGGTAGCC<br>CAGTGAACGAGCAGAACCTGTG
VTL4F (JW65)<br>VTL4R (JW83)	Genotyping of *vtl4* mutants	CAACCTCCAACCACCACTCT<br>TGTACTTCATGGCTTCTCTTGG
JW3<br>JW4	RT-PCR of *VTL4*	AGCTGCTTTGTTAGGAGCCA<br>ATGTCTTCATGCACAGCTCCA
JW98<br>JW99	RT-PCR of *VTL8*	GATTATTGGCAAAGGGCTCA<br>ATAGCCATAGCCATCCATCCACCAAG
JW5<br>JW6	RT-PCR of *ACTIN* (Medtr3g095530)	TCAATGTGCCTGCCATGTATGT<br>ACTCACACCGTCACCAGAATCC
VTL4RTqF<br>VTL4RTqR	RT-qPCR of *VTL4*	ACCACCAGAAATGAGAAAGAGC<br>TTGAAGTTGGCCACCTCTGA
SEN1qRTF<br>SEN1qRTR	RT-qPCR of *VTL8*	GGCTTTGGTTGTGTTTGGAGG<br>GCCATAGCCATCCATCCACC
UPL7qRTF<br>UPL7qRTR	RT-qPCR reference gene<br>(Medtr7g103210)	CCAGTTGTTCTCGTGGTCCATT<br>CCTCCAATTGTCGCCCAAA
mbfAQF<br>mbfAQR	RT-qPCR of *mbfA*	AAGAGGACGATGGACGCATC<br>CTCTCCTCCTCCGCCATTTC
gapAQF<br>gapAQR	RT-qPCR reference gene<br>(*S. meliloti gapA*, SMc03979)	GAAATCAACGCGGCCATCAA<br>CGGATCGTGGTTGAAGTCGA
FL3	Linker peptide<br>LGGGGSGGGGSGGGGSAAA	CTTGGTGGAGGTGGATCTGGTGGAGGTGG<br>ATCAGGTGGAGGTGGATCTGCTGCAGCT
	Cloning of *MtVTL4* into pYES2<br>(Two-step PCR for Gateway)	AAAAAGCAGGCTCCATGGCTTCTCTTGGTAACCATAAT<br>AGAAAGCTGGGTATCAAAGTGAACTATAGCCAACGAA
M39<br>M40	Cloning of *MtVTL8* into pYES2<br>(One-step PCR for Gateway)	ggggacaagtttgtacaaaaaagcaggctcgATGGCCGTTGGTACAATATG<br>ggggaccactttgtacaagaaagctgggtaTCAAATTTCCAAATCCAAACC
	Cloning of *AtVIT1* into pYES2<br>(One-step PCR for Gateway)	ggggacaagtttgtacaaaaaagcaggcttaATGTCGTCGGAGGAAGATAAGA<br>ggggaccactttgtacaagaaagctgggtaCTAATGTTGCACAACTTTAGCC
mbfANEWF<br>mbfANEWR	Cloning of *S. meliloti mbfA* promoter	ACTGGGTACCGACAAGGTGGTGGCGATGTA<br>ACTGGGATCCAATGCGTCCATCGTCCTCTTC
ICESDM2F	Mutagenesis of *mbfA* ICE box	CATCACGAAGCCTTCTGAAGCTTAATTCTAATTAGGCGCCGATTCATGACGCG

ICESDM2R		CGCGTCATGAATCGGCGCCTAATTAGAATTAAGCTTCAGAAGGCTTCGTGATG
KGWSpeI_325pr_F<br>KGWAatII_325UTR_R	Cloning of *VTL4* into vector pKGW-R	aggcggccgcactagTCGTGACGGAAAACATGTAGGG<br>gcaccaaccacaacgacgtTGAGCTGAATTTTTGAGCTGACTATAC
KGWSpeI_335pr_F<br>KGWAatII_335UTR_R	Cloning of *VTL8* into vector pKGW-R	aggcggccgcactagCCAAACGACATGGCATATAGC<br>gcaccaaccacaacgacgtCATATAAACCTCCCGAAACCCTC
325prom_F<br>335_R&325prom_hom	Cloning of *VTL8* + *VTL4* into vector pKGW-R	TCGTGACGGAAAACATGTAGGG<br>TGTTTTCCGTCACGACATATAAACCTCCCGAAACCCTC
h2_16e5_F<br>h2_16e5_R<br>h2_17n4b_F<br>h2_17n4b_R<br>h2_68o3b_F<br>h2_68o3b_R<br>mth2-49e15_F<br>mth2-49e15_R<br>mth2_72d18e_F<br>mth2_72d18e_R	Fine mapping of 13U	CTGCAATTAAACAAAATGGTTAGAGT<br>CTACCAAACAAAAGCCTAGACTTCTT<br>CAACGCCGGGTAAATACACT<br>TTGTTTTTGTGTTTCGCACA<br>GGTTCAACAATCAAATGAGCTG<br>GTGAGCAGAAGCATACACGC<br>AACCCCAGACACCCCACT<br>GCACGCATGCACACTCAT<br>TGACCAATGAAGCGCAATAC<br>TATTAAAACATCCCGACCGC
BG646574_F2<br>BG646574_R2<br>SYN24_F<br>SYN24_R<br>TC173960_F1<br>TC173960_R1b<br>TC178276_F1<br>TC178276_R1<br>TC182270_F1<br>TC182270_R1	Delimiting the 13U deletion	GAGTCAAACGAAGTAAAGGTT<br>ACTCAAAACTGGAACTCTGAC<br>TCAACTCAACAATGCTTACTATGGC<br>AAAGAGGAAACCAATGATTGCTACT<br>TCACCATTGCCCCACTGATGCTA<br>ACAACCTTATTCCAGGAGTTACC<br>GGCAAAGGGCTCAATGGCTTCGC<br>GCACCAACACCTCCAAACACAAC<br>TTCTTTGCCTTTTTATTTTTCGTTC<br>TGGTTCAGGTCCTTCATTAGTCTTG
TC192817_F1<br>TC192817_R1<br>CA919468_F1<br>CA919468_R1<br>TC191603_F1<br>TC191603_R1	Detecting borders of the 13U deletion	CAACAAACCAAAAGACCAGCATAGG<br>AGGGATGGTGGTAGTCGTGGAGATT<br>AATGAATGACCCTATCTCACGGA<br>ATTTCTACATCTGCACTTGGCTT<br>TCAGGCTTCTCACTATGGCTTATT<br>CACTGACCCAGAGACCACAATACC

Table S3 Expression of *S. meliloti* genes involved in Fe homeostasis during nodule development
Data from Roux B *et al*. 2014. An integrated analysis of plant and bacterial gene expression in symbiotic root nodules using laser-capture microdissection coupled to RNA sequencing. *Plant Journal* **77**: 817–837.

		Total reads	Percentage (%) expression				
Medicago truncatula			FI (zone I)	FIId	FIIp	IZ	ZIII
VTL4	Medtr4g094325	40662.4	3.9	44.1	8.6	16.7	26.8
VTL8	Medtr4g094335	150938.1	0.1	0.2	7.3	68.6	23.8
Sinorhizobium meliloti							
bfr (putative bacterioferritin)	SMc03786	6959.4	21.6	24.6	22.7	26.2	4.9
fecI (Fe citrate transport, regulation)	SMc04203	188.5	13.8	16.5	32.2	21.2	16.3
fecR	SMc04204	643.0	2.0	11.4	40.6	24.3	21.7
hmuV (hemin uptake)	SMc01510	556.0	20.5	30.5	25.3	11.0	12.9
hmuU	SMc01511	1240.2	24.3	26.1	27.8	11.9	9.9
hmuT	SMc01512	1655.5	25.4	22.1	33.5	14.2	4.9
hmuS	SMc01513	3746.6	32.6	18.4	29.9	15.3	3.8
Irr (iron response regulator)	SMc00329	1122.6	12.9	17.8	31.8	30.0	7.6
mbfA (membrane-bound ferritin)	SMc00359	6289.4	1.9	3.0	38.7	48.0	8.4
rhbA (rhizobactin biosynthesis)	SMa2400	391.5	70.0	4.6	5.3	5.6	14.5
rhbB	SMa2402	391.5	70.0	4.6	5.3	5.6	14.5
rhbC	SMa2404	961.7	32.3	17.5	25.2	15.1	10.0
rhbD	SMa2406	515.2	49.8	11.6	8.6	12.2	17.8
rhbE	SMa2408	131.6	66.0	7.2	8.0	7.9	10.8
rhbF	SMa2410	955.8	26.4	14.7	14.9	8.9	35.1
rhtA (rhizobactin transporter)	SMa2414	3406.1	65.0	10.6	4.9	6.2	13.3
Putative Fe/heme transporter	SMc04205	461.9	11.1	8.6	16.0	23.4	41.0

Methods S1 Additional information on Materials and Methods

The *Medicago truncatula* Vacuolar iron Transporter-Like proteins VTL4 and VTL8 deliver iron to symbiotic bacteria at different stages of the infection process

Authors: Jennifer H. Walton, Gyöngyi Kontra-Kováts, Robert T. Green, Ágota Domonkos, Beatrix Horváth, Ella M. Brear, Marina Franceschetti, Péter Kaló and Janneke Balk

Gene expression analysis

Total RNA from plant tissue was extracted using the RNeasy Mini kit (QIAGEN, Germany) and treated with DNase (Turbo DNase kit, Agilent). RNA from bacteria was isolated using RNAprotect Bacteria Reagent and the RNeasy Mini kit, both from QIAGEN, following manufacturer's protocols. cDNA was produced using Thermo SuperScript II Reverse Transcriptase and an anchored oligo-dT primer, and used as template for either standard RT-PCR with products separated by agarose gel electrophoresis, or for quantitative RT-qPCR. RT-qPCR reactions were made using SensiFAST master-mix (Bioline), each with 20 ng of cDNA. Reactions were measured in a Bio-Rad CFX-96 real-time PCR system and cycled as per the Bioline protocol. The expression values of *VTL4* and *VTL8* were normalized to that of the *UPL7* gene (*UBIQUITIN PROTEIN LIGASE 7*), and the expression of *mbfA* was normalized to *gapA* expression, see Table S2 for primers.

Acetylene reduction assay

The activity of nitrogenase was assayed by testing the capacity of nodules to reduce acetylene to ethylene, another reaction catalyzed by the enzyme. Nodulated roots were harvested 28 dpi and those of two or three single plants were placed in 1.8 ml glass vials sealed with a rubber cap. To start with, dilutions of ~99.5% ethylene were injected into a Shimadzu 2010 GC gas chromatograph using a HP-PLOTQ (30 m x 320 μm x 20 μm) column to generate a calibration curve. Next, 180 μl of acetylene was injected into each vial containing detached nodulated roots. After incubation at room temperature for at least 2 h, 100 μl gas samples were taken and injected into the gas chromatograph to determine the amount of ethylene produced. After the assay, nodules were picked off the roots and

weighed. The acetylene reduction activity (ARA) was calculated as picomole of ethylene per min per mg nodule weight.

Measuring bacteroid length

About 80 mg nodules were homogenized in 300 μL ice-cold PBS, pH 7.4. Cell debris was removed by centrifugation (500 x *g*, 10 min, 4°C) and supernatant was filtered through a CellTrics nylon filter of mesh size 50 μm (Sysmex,Partec GmbH Görlitz, Germany). The flow-through was filtered again through 20 μm mesh sized nylon and bacterial cells were pelleted by centrifugation (5000 x *g*, 15 min, 4°C). The pellet was washed and suspended in 20 μL PBS. 3 μL suspensions of bacteria were dropped on microscope slides and 1 μL propidium-iodide (20 μM) was added. Following 1 h incubation, at least five images per line were taken with a Leica TCS SP8 confocal laser scanning microscope with objective lens: HCX PL FLUOTAR 10x/0.30 (dry, NA:0.3), PL FLUOTAR 40x/1.00 OIL. The detection range for the PI channel was 561-660 nm. The length of ≥ 2500 bacterial cells from wild-type R108 and mutant *vtl4* alleles; ≥ 800 from wild-type Jemalong and mutant 13U; and ≥ 400 from the *dnf7-2* mutant (Horvath *et al.*, 2015) was measured using ImageJ software. The relative distribution of different bacteroid size was plotted.

Yeast complementation

The yeast strain DY150, which is derived from W303, was used as wild type. The Δ*ccc1* strain in this background carries a genomic deletion of *CCC1*, initially identified as *Cross-Complements* Ca^{2+}*1*, but later shown to mediate vacuolar iron transport (Li *et al.*, 2001). Plant genes were cloned into shuttle vector pYES2 under the control of the *GAL1* promoter for galactose-inducible expression. The coding sequence of Arabidopsis *VIT1* (*AT2G01770*) was used as a positive control for functional complementation (Kim *et al.*, 2006). Yeast were transformed using the lithium-acetate method and positive transformants were selected on synthetic dropout medium lacking uracil (DSCK102, Formedium, Hunstanton UK) with glucose as carbon source (SD). Overnight cultures of selected colonies were grown in SD-Ura, then spotted onto 2% (w/v) agar plates of SGal-Ura with or without 5 mM $FeSO_4$. Plates were photographed after 4 days (control) or 5 days (with iron).

Iron measurement and Perls' staining

To measure total iron, nodules were dried and mineralized in 1:1 volumes of nitric acid and hydrogen peroxide (30%), followed by quantitative analysis using the colorimetric chelator ferene (3-(2-pyridyl)-5,6(5-sulfo-2-furyl)-1,2,4-triazine). Iron staining of nodules was performed using the Perls' method, which stains non-haem iron (Meguro *et al.*, 2007). In brief, nodules were fixed in 4% (w/v) paraformaldehyde, then incubated with 1:1 volumes of 4% (w/v) potassium ferrocyanide and 4% (w/v) HCl for 1 h and washed with water. Wild-type nodules were additionally treated with 1:1 volumes of 4% (w/v) potassium ferricyanide and 4% (w/v) HCl for 40 h to stain haem iron in the infected cells. Tissue samples were embedded in 5% (w/v) agarose and sectioned with a VT1200 vibratome (Leica). Images of the mounted sections were taken using a LEICA DM6000 microscope, with x20/0.7 air or x40/0.85 air objectives and a LEICA DFC420C colour camera.

Protein blot analysis

Plant tissue (~10 mg) was homogenised with 0.4 ml of 10% (w/v) trichloroacetic acid in acetone at 4°C. After incubation at -20°C for 1 h, the sample was centrifuged at 16,000 x *g,* 4°C, for 10 min. The pellet was washed 3 times with cold acetone, dried and dissolved in 50 µl Laemmli buffer (125 mM Tris-HCl pH 6.8, 2% (w/v) sodium dodecyl sulphate, 10% (v/v) glycerol, 5% (v/v) 2-mercaptoethanol and 0.04% (w/v) bromophenol blue). Samples (10 µl) were separated by SDS-PAGE and transferred under semi-dry conditions to nitrocellulose membrane. Ponceau-S staining of the membranes was used to confirm equal protein loading and successful transfer. The membrane was blocked in Tris-buffered saline (TBS) containing 0.1% (v/v) Tween-20 and 5% (w/v) skimmed dried milk (TBS-TM) for 1 h. Antibodies were diluted 1:2000 – 1:5000 in TBS-TM and incubated with the membrane for 1 – 2 h. Membranes were washed in TBS-TM for 3 x 5 min, then incubated with horseradish peroxidase-conjugated anti-rabbit IgG for 45 min. After washing 4 x 5 min in TBS-T, the positive immunosignal was developed using ECL reagent and exposed to Amersham Hyperfilm MP (GE Healthcare). Polyclonal antibodies against *Pisum sativum* ferritin (product number AS15 2898) and Arabidopsis hemoglobin 2

(AHB2, product number AS13 2745) were from Agrisera, Vännas, Sweden. Both antibodies are known to cross-react with their target in a range of other plant species.

References

Horvath B, Domonkos A, Kereszt A, Szucs A, Abraham E, Ayaydin F, Boka K, Chen Y, Chen R, Murray JD, *et al.* 2015. Loss of the nodule-specific cysteine rich peptide, NCR169, abolishes symbiotic nitrogen fixation in the Medicago truncatula dnf7 mutant. *Proceedings of the National Academy of Sciences, USA* **112**: 15232–15237.

Kim S, Punshon T, Lanzirotti A, Li LT, Alonso JM, Ecker JR, Kaplan J, Guerinot ML. **2006**. Localization of iron in *Arabidopsis* seeds requires the vacuolar membrane transporter VIT1. *Science* **314**: 1295–1298.

Li L, Chen O, McVey Ward D, Kaplan J. **2001**. CCC1 is a transporter that mediates vacuolar iron storage in yeast. *Journal of Biological Chemistry* **276**: 29515–29519.

Meguro R, Asoano Y, Odagiri S, Li C, Iwatsuki H, Shoumura K. **2007**. Nonheme-iron histochemistry for light and electron microscopy: a historical, theoretical and technical review. *Archives of Histology and Cytology* **70**: 1–19.
